# Differential expression of sirtuin family members in the developing, adult, and aged rat brain

**DOI:** 10.3389/fnagi.2014.00333

**Published:** 2014-12-18

**Authors:** Elena Sidorova-Darmos, Robert G. Wither, Natalya Shulyakova, Carl Fisher, Melanie Ratnam, Michelle Aarts, Lothar Lilge, Philippe P. Monnier, James H. Eubanks

**Affiliations:** ^1^Division of Genetics and Development, Toronto Western Research InstituteToronto, ON, Canada; ^2^Department of Physiology, University of TorontoToronto, ON, Canada; ^3^Department of Medical Biophysics, University of TorontoToronto, ON, Canada; ^4^Department of Cell Systems Biology, University of TorontoToronto, ON, Canada; ^5^Institute of Medical Sciences, University of TorontoToronto, ON, Canada; ^6^Department of Surgery (Neurosurgery), University of TorontoToronto, ON, Canada

**Keywords:** sirtuin, aging, astrocyte, neuron, expression, development, immunoblot, real time PCR

## Abstract

The sirtuins are NAD^+^-dependent protein deacetylases and/or ADP-ribosyltransferases that play roles in metabolic homeostasis, stress response and potentially aging. This enzyme family resides in different subcellular compartments, and acts on a number of different targets in the nucleus, cytoplasm and in the mitochondria. Despite their recognized ability to regulate metabolic processes, the roles played by specific sirtuins in the brain—the most energy demanding tissue in the body—remains less well investigated and understood. In the present study, we examined the regional mRNA and protein expression patterns of individual sirtuin family members in the developing, adult, and aged rat brain. Our results show that while each sirtuin is expressed in the brain at each of these different stages, they display unique spatial and temporal expression patterns within the brain. Further, for specific members of the family, the protein expression profile did not coincide with their respective mRNA expression profile. Moreover, using primary cultures enriched for neurons and astrocytes respectively, we found that specific sirtuin members display preferential neural lineage expression. Collectively, these results provide the first composite illustration that sirtuin family members display differential expression patterns in the brain, and provide evidence that specific sirtuins could potentially be targeted to achieve cell-type selective effects within the brain.

## Introduction

The family of mammalian sirtuins consists of seven members (SIRTs 1-7), which collectively comprise the class III protein deacetylases (reviewed in Michan and Sinclair, 2007). These sirtuins are descendants from the ancestral yeast gene *Sir2*, which was originally identified as a silent mating factor (Frye, 2000; Greiss and Gartner, 2009). These mammalian sirtuins function as NAD^+^-dependent protein deacetylases and/or ADP-ribosylases that exert their actions largely in distinct subcellular compartments. SIRT1, SIRT6, and SIRT7 are found primarily within the nucleus, where they target proteins in nucleoplasm, along heterochromatin and associated with nucleoli, respectively (Michishita et al., 2005). Although predominantly nuclear, there is also evidence that at least SIRT1 can shuttle to the cytosol under certain conditions (Michishita et al., 2005; Jin et al., 2007). In contrast, SIRT3, SIRT4, and SIRT5 localize primarily within mitochondria (Onyango et al., 2002; Ahuja et al., 2007; Nakagawa et al., 2009), while SIRT2 is predominately found in the cytoplasmic compartment of cells (North et al., 2003). Converging evidence has implicated the function of specific sirtuin family members in different aspects of cellular regulation, including lipid homeostasis and metabolism (reviewed in Houtkooper et al., 2012), oxidative stress management (reviewed in Rajendran et al., 2011), and apoptosis resistance (reviewed in Pantazi et al., 2013). As these sirtuin targets are believed to contribute to aging processes, there is speculation that sirtuin family members may play direct roles in longevity regulation (Guarente, 2013). The catalytic activity of the different sirtuins increases or decreases depending on factors such as the availability of NAD^+^, levels of cellular stress, glucose availability, oxidative load, and/or the energy production demands within the individual cell (Pillai et al., 2005; Yang et al., 2007; Ying and Xiong, 2010; Braidy et al., 2011; Zerp et al., 2014). Thus, sirtuins appear to serve as metabolic and stress sensors whose activity can regulate the homeostatic balance of cells in response to changing environmental conditions.

Despite these data illustrating the prominent role of sirtuins in cellular homeostasis, relatively little information currently exists regarding their expression magnitude and spatial distribution within the central nervous system. Given that the brain utilizes approximately 20% of total body energy expenditure (reviewed in Herculano-Houzel, 2011), an understanding of sirtuin expression in this metabolically active tissue could aid in delineating their role in both normal physiological processes and also potentially in pathophysiological conditions. To begin addressing this issue, we examined the endogenous mRNA and protein expression patterns of the seven sirtuin family members in the adult, developing and aged rat brain, and compared their relative expression within the two predominant cell types of the brain, specifically neurons and astrocytes. Our results indicated that while each sirtuin is expressed within the brain, there are dramatic differences between their expression patterns at the spatial and ontogenetic levels.

## Materials and methods

### Animal use and tissue collection

Animal experimentation was conducted in accordance with the guidelines of the Canadian Council of Animal Care, and protocols for animal use were reviewed and approved by the local University Health Network animal care committee before initiation of the study. Male Wistar rats and timed pregnant female Wistar rats were purchased from Charles River Laboratories (Sherbrooke, Quebec). SIRT3-null mice and wild-type mice were obtained from Jackson Laboratories (SIRT3-KO mice, cat # 012755, wild-type mice, cat #000664; Bar Harbor, ME). Cortical tissue was harvested from these mice for SIRT3 antibody validation. For expression analysis Wistar rats were used as subjects. These subjects were euthanized by decapitation while under isoflurane anesthesia, and specific brain and peripheral tissues were collected by dissection (whole brain, cortex, hippocampus, cerebellum, brain stem, spinal cord, striatum, olfactory bulb, heart, liver, spleen, kidney, small intestine) at different ages (embryonic day 18 (E18), postnatal day (PN) 2, 7, 21 and 3, and 24 months). The gender of the subjects used for tissue collection was not specifically determined for E18, PN2, PN7 or PN21 stages, while female rats were used for the 3 and 24 month tissue samples. These dissections were conducted on an ice-cold glass plate, and the isolated tissues snap frozen by submersion in liquid nitrogen. Frozen tissues were stored at −80°C until homogenization for RNA or protein examinations.

### Establishment of neuron-enriched primary cortical cultures

Cortical tissue was isolated from fetuses obtained at embryonic day 18 (E18) from timed-pregnant Wistar rats (Charles River Laboratories). The head of each embryo was dissected away from the body, and placed in 20 ml 1× Hank's Balanced Salt Solution (HBSS) (Invitrogen-Life Technologies, Burlington, Ontario, cat # 14175103). Brains were removed and placed in a separate dish containing 20 ml supplemented HBSS. Cortices were dissected from whole brains using micro dissection forceps, and the collected tissue was incubated in 2 ml of 0.05% trypsin (Sigma–Aldrich, St. Louis, MO, cat # 59417C) at 37°C for 15 min. The tissue was then triturated by glass pipette 10–15 times to disperse the cells in seeding medium (Dulbecco's Modified Eagle Medium (DMEM) /F-12) (Invitrogen, cat # 21041025) containing 10% HS and then centrifuged for 5 min at 21,000 × g to pellet the cells. The cells were re-suspended in Neurobasal medium (Invitrogen, cat # 21103049) containing 2% B-27 supplement (Invitrogen, cat # 17504-044), 1% FBS, 0.5 mM L-glutamine (Invitrogen, cat # 25030-149) and cell counts done using Trypan Blue (Sigma–Aldrich, cat # T8154) as a viability indicator. The cells were then seeded in plating medium at a density of 30,000 cells/well in 96-well plates. After 96 h of isolation, the plating media was exchanged with maintenance medium (Neurobasal medium containing 2% B-27 supplement, 0.5 mM L-Glutamine) containing 4 μM cytosine arabinoside (Sigma-Aldrich, cat # C6645) to inhibit proliferation of any residual non-neuronal cells. The primary cultures were fed with maintenance medium every 3–4 days, and the neurons were harvested for assay at 12–14 days after plating.

### Preparation of astrocyte-enriched cultures

Astrocyte enriched cultures were generated from cortical tissue isolated from post-natal day 1-2 rat pups. In short, following decapitation, cortical tissue was isolated by dissection, placed in ice-cold HBSS, and the meninges removed by dissection. The tissue was then rinsed in HBSS, placed in a minimal amount of media, and chopped into 1 mm^3^ cubes by manual dissection. The brain cubes were then incubated with 0.05% Trypsin for 30 min at 37°C, and dissociated by triturating 12–15 times with polished Pasteur pipette. The cells were then collected by centrifugation at 21,000 × g for 5 min, re-suspended in Astrocyte Medium containing 1× N2 Supplement (Invitrogen, cat # 17502048), 2 mM Glutamax, Penicillin/Streptomycin, supplemented with 5 ng/ml EGF (Sigma-Aldrich, cat # SRP3052) as per manufacturer's instruction. Trypan Blue was used to count viable cells and the cell suspension was then plated in at a density of 1,000,000 cells/well in 6-well plates.

### Quantitative real time PCR

To quantify sirtuin mRNA expression levels, rats were sacrificed by decapitation, and specific brain regions isolated by dissection on an ice-cold glass plate. RNeasy Mini Kit (Qiagen, Valencia, CA, cat # 74104) was used to extract total RNA from the tissue as per manufacturer's guidelines. Purified total RNA (1 μg) was then reverse transcribed into cDNA using the Superscript II First Strand Synthesis System (cat # 18064-014, Invitrogen). Pre-designed Taqman probe and primer mixtures (Applied Biosystems-Life Technologies, Burlington, Ontario) were used to assess mRNA expression: *Sirt2* (cat # Rn01457502_m1), *Sirt3* (cat # Rn01501412_m1), *Sirt4* (cat # Rn01481485_m1), *Sirt5* (cat # Rn01450559_m1), *Sirt6* (cat # Rn01408249_m1), and *Sirt7* (cat # Rn01471420_m1), using hypoxanthine-guanine phosphoribosyltransferase (*Hprt1*) (cat # Rn_01527831_g1) as a reference control. Because Taqman primers were not available for rat *Sirt1*, SYBR Green forward and reverse primers were designed that spanned an intron of the rat Sirt1 sequence (forward: 5′-cagcaacacctcatgattgg-3′, reverse: 5′-tcccacaggaaacagaaacc-3′) and also for the reference *Hprt1* (forward: 5′-gcagactttgctttccttgg-3′, reverse: 5′-cgagaggtccttttcaccag-3′). We selected *Hprt1* as the housekeeping gene for our mRNA quantification studies as it is expressed ubiquitously and stably in various tissues (Fischer et al., 2005; Wei et al., 2013), and it has been used as an internal control for mRNA normalization in previous developmental studies (Franco-Montoya et al., 2011; Murta et al., 2013). All PCR samples were assayed in quadruplicate on a 384-well plate using Taqman master mix reagent kit (Applied Biosystems, cat # 4304437) or SYBR Green (Applied Biosystems, cat # 4368577) using 20 ng of cDNA generated from total isolated RNA as template in 10 μl reactions on a 7900HT Fast Real-Time PCR System (Applied Biosystems). The thermal profile for the Taqman assays was as follows: one cycle of enzyme activation at 50°C for 2 min and template denaturation at 95°C for 10 min, followed by 40 cycles of 95°C for 15 s, primer annealing at 60°C for 1 min, and template extension at 72°C for 30 s. For SYBR Green assays, the thermal profile was: one cycle of enzyme activation at 50°C for 2 min followed by denaturation at 95°C for 10 min, followed by 40 cycles of 95°C for 15 s, primer annealing at 56 or 60°C for 1 min, and template extension at 72°C for 30 s. The amplification of all the primers was analyzed with using the Applied Biosystems Sequence Detection System software (SDS v. 2.2). For mRNA expression, critical threshold (Ct) cycle values were identified for each sirtuin being assayed relative the expression of the *Hprt1* reference gene, and the results expressed as fold differences from *Hprt1* using the 2^−ΔCt^ formula, where ΔC_t_ = C_t_(*Sirt*) − C_t_ (*Control*). For mRNA expression comparisons between the astrocyte and neuronal enriched cultures, the expression of *Hprt1* was used as a common reference. The Ct values for *Hprt1* expression were equivalent between the two cultures (not shown).

### Antibodies and western blotting

Following harvest, cultured cells were homogenized in tissue lysis buffer consisting of 50 mM Tris—pH 8.0, 1% NP40, 150 mM NaCl, 1mM EDTA, 1mM PMSF, 1 μg/ml Aprotinin, 1 μg/ml Leupeptin, 2 mM Na_3_VO_4_ and supplemented with 1 tablet of protease inhibitor cocktail (Roche, cat # Mississauga, Ontario, cat # 11836153001). Frozen tissue was minced while still lightly frozen and homogenized as above in lysis buffer. Following homogenization through a 21 gauge needle, the samples were centrifuged at 21,000 × g for 10 min at 4°C. The supernatant was then collected, and total protein concentration determined using the Bio-Rad protein assay (Biorad, Mississauga, Ontario, cat # 162-0115). For immunoblots, 20 μg of total protein samples were resolved on 12% SDS-PAGE, at a constant voltage of 100 V for 2 h. The proteins resolved in the gel were then transferred to PVDF membranes (PALL, Mississauga, Ontario, cat # 66543) overnight in transfer buffer (25 mM Tris, 192 mM glycine, and 0.1% SDS) at 4°C at a constant voltage of 25 V. The membranes were then blocked for 2 h in non-fat powdered milk (5%) in TBST washing buffer (10 mM Tris, 150 mM NaCl, 0.05% Tween 20), and probed overnight at 4°C with a the primary antibody of interest. The antibodies and dilutions used are: rabbit polyclonal anti-SIRT1 (1:10,000; Millipore, Billerica, MA, cat # 07-131); rabbit polyclonal anti-SIRT2 (1:2000; Sigma-Aldrich, cat # S8447); rabbit monoclonal anti-SIRT3 (1:500; Cell Signaling, Beverly, MA, cat # 2627); rabbit monoclonal anti-SIRT3 (1:500; Cell Signaling, cat # 5490), rabbit polyclonal anti-SIRT4 (1:1000; Sigma–Aldrich, cat # S0948); rabbit polyclonal anti-SIRT5 (1:500; Millipore, cat # ABE198); rabbit polyclonal anti-SIRT6 (1:1000; Sigma-Aldrich, cat # S4322); rabbit polyclonal anti-SIRT7 (1:250 to 1:3000; Sigma-Aldrich, cat # AV32406); mouse monoclonal anti-glial fibrillary acidic protein (GFAP) (1:15,000; Cell Signaling, cat # 3670); mouse monoclonal anti-microtubule associated protein 2 (MAP2) (1:500; Sigma–Aldrich, cat # M1406); mouse monoclonal anti-NG2 (1:500; Chemicon-Millipore, cat # MAB5384). After washing in TBST 3 times for 15 min each, the blots were incubated with the appropriate horseradish peroxidase (HRP)-linked secondary anti-rabbit raised in sheep (1:5000; GE Healthcare, cat # NA934V) or anti-mouse raised in donkey (1:5000; GE Healthcare, cat # NA931V) antibodies at room temperature for 2 h, and washed in TBST as above. Immunoreactivity was visualized using Western-Lightning Chemiluminescence Reagent Plus (PerkinElmer, Waltham, MA, cat # NEL-105) on X-Ray film (Denville Scientific, Metuchin, NJ, cat # E-3018). To allow normalization for minor variances in protein loading between sample lanes, the blots were re-probed for anti-glyceraldehyde-3-phosphate dehydrogenase (GAPDH) (1:5000; Chemicon-Millipore cat # MAB374), Beta-actin (1:1000; Cell Signaling, cat # 51525) or stained with Coomasie blue (0.1% Coomassie Brilliant Blue R250, 10% acetic acid, Sigma-Aldrich). Coomasie staining was used as the normalization control for examination of peripheral tissue expression levels. This latter means of normalization was required, as classic loading controls such as GAPDH have not proven reliable when comparing across different tissues and/or specific cell types from the same animal (Eaton et al., 2013). Quantity One software (BioRad) was used to analyze scanned Autoradiography films (Denville Scientific, cat # E3018). Optical densities of all bands were measured in OD/mm^2^, with film background subtracted to give the final densitometric values.

### Validation of antibody specificity

PC12 cells were grown in at 37°C in a humidified incubator under 5% CO_2_ conditions. The culture medium was RPMI 1640 (Gibco-Life Technologies, Burlington, ON, cat # 11875-093), supplemented with 10% horse serum (HS) (Gibco, cat # 16050-122), 5% fetal bovine serum (FBS) (Gibco, cat # 16000-044), and 50 U/ml penicillin-streptomycin (Gibco, cat # 15240-062). The specificity of each individual sirtuin antibody was determined by assessing non-treated and siRNA-treated PC12 cells for the appropriate sirtuin. The specific siRNAs and concentrations used were: *Sirt1* (50 nM, Sigma-Aldrich, cat # SASI_Rn0200230695), *Sirt2* (13 nM, Ambion-Life Technologies, Burlington, Ontario, cat # S16750), *Sirt4* (13 nM, Ambion, cat # S156091), *Sirt5* (50 nM, Ambion, cat # S157785), *Sirt6* (50 nM, Ambion, cat # S152314), *Sirt7* (50–200 nM, Ambion, cat # S155507), and a scrambled siRNA control at matching concentrations (Ambion, cat # AM4611) was used as a negative control for each respective comparison. After 24 h of transfection, whole cell lysates were collected and analyzed by western blotting. For SIRT3, cortical tissue from SIRT3-KO mice was used to confirm antibody specificity, as PC12 cells did not display readily discernible endogenous SIRT3 immunoreactivity under basal conditions (not shown). Cortical tissue was isolated and processed as described above.

### Statistical analysis

Statistical analysis was performed using either student's *t*-test or One-Way ANOVA in GraphPad Prism version 5.00 (GraphPad Software, San Diego CA), with Tukey's *post-hoc* test for multiple comparisons where appropriate. All results are expressed as mean ± standard error of means (SEM) and *p* < 0.05 was set as the level of statistical significance.

## Results

### Sirt1

**Figure 1:**
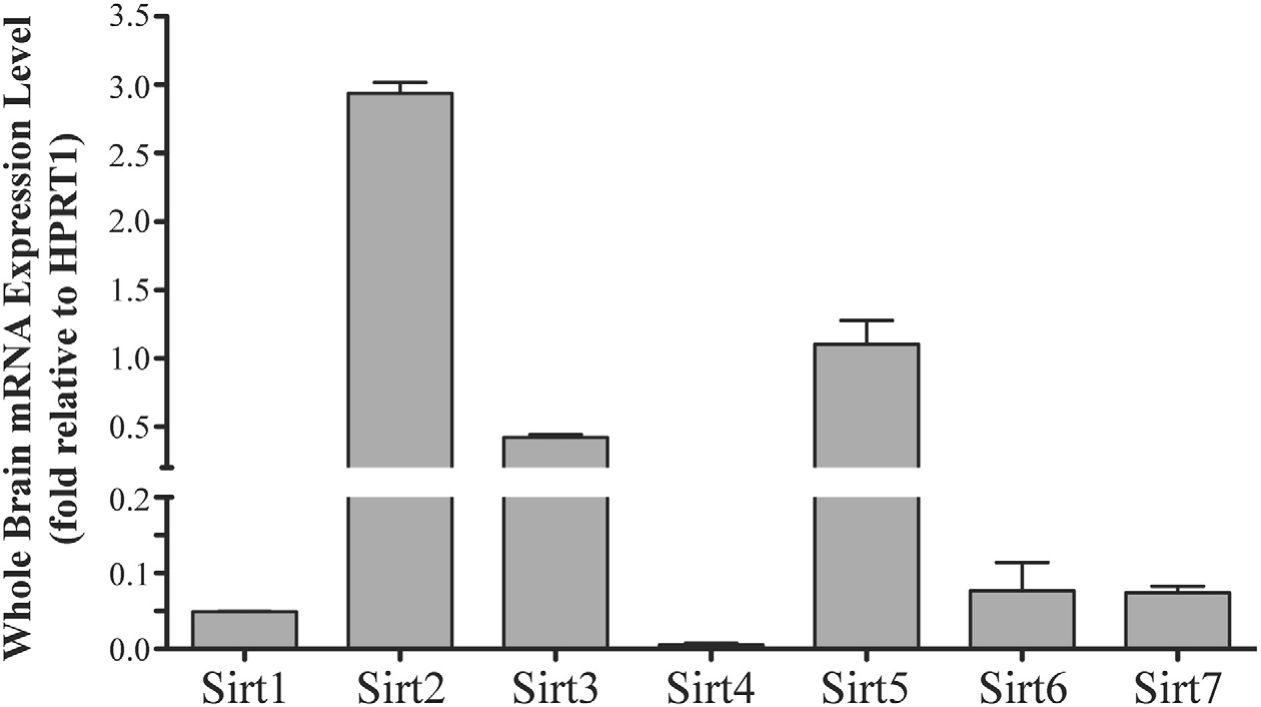
**Sirtuins are expressed at different levels in the adult rat brain**. Histogram showing *Sirt1-7* mRNA expression levels in the whole adult rat brain as determined by qRT-PCR. The y-axis shows the relative sirtuin expression levels relative to the *Hprt1* reference gene. Note the broken y-axis scale for Sirt2, Sirt3 and Sirt5. Data shown are based on the linear conversion of delta CT values for each sample (*n* = 3, error bars denote SEM). Statistical comparisons between the expression levels of the different sirtuins in the adult brain are presented in Supplemental Table 1.

**Figure 2:**
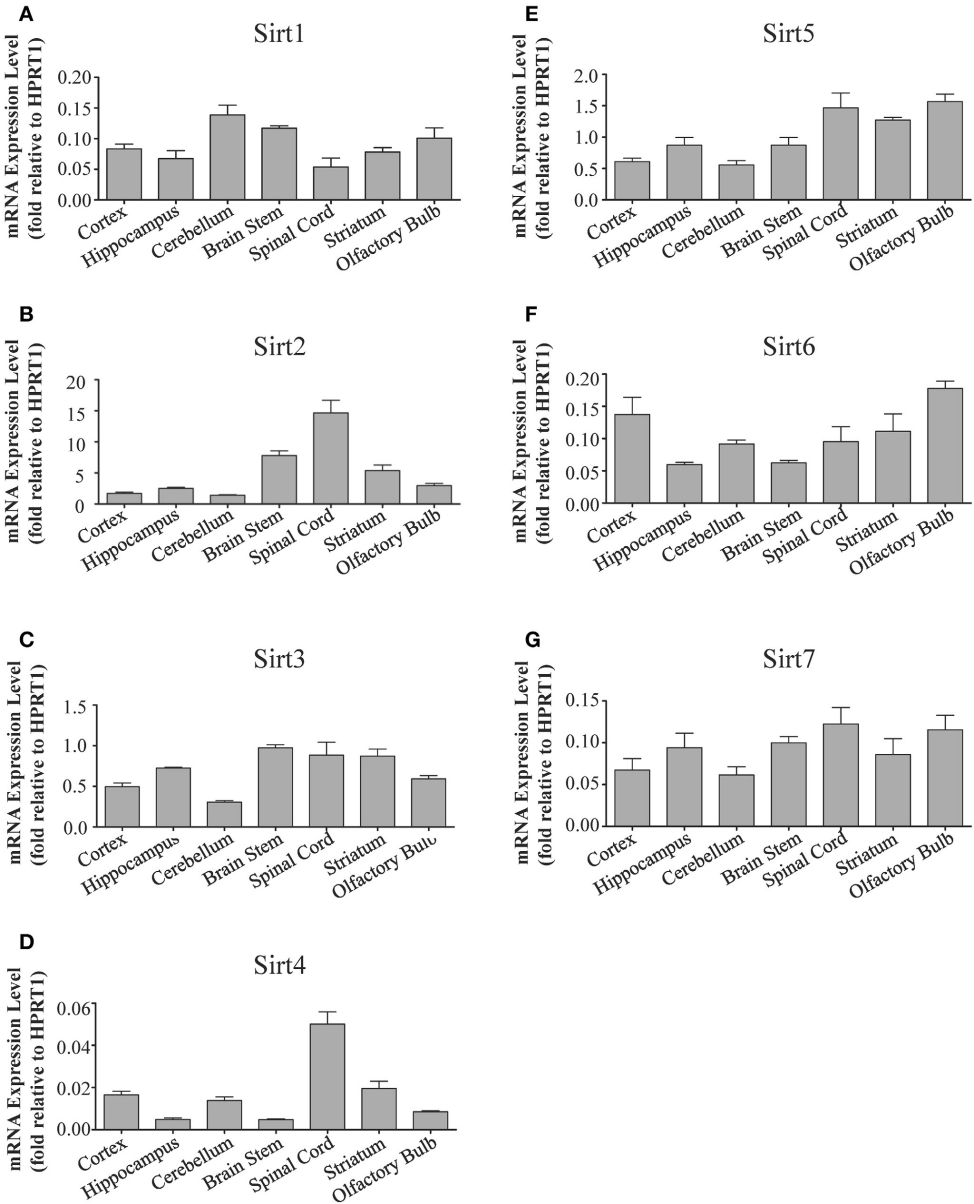
**Sirtuins display differential gene expression levels within different regions of the adult rat brain**. Histograms showing the mean and SEM of the mRNA expression levels for each individual sirtuin in the indicated brain regions as determined by qRT-PCR. The y-axis shows the mRNA expression levels for **(A)** *Sirt1* **(B)** *Sirt2* **(C)** *Sirt3* **(D)** *Sirt4* **(E)** *Sirt5* **(F)** *Sirt6* **(G)** *Sirt7* relative to the *Hprt1* reference gene in each of the denoted brain regions. Data shown are based on the linear conversion of delta CT values for each sample (*n* = 3, error bars denote SEM). Statistical comparisons for the expression levels of each sirtuin in these adult brain regions are presented in Supplemental Table 2.

**Figure 3:**
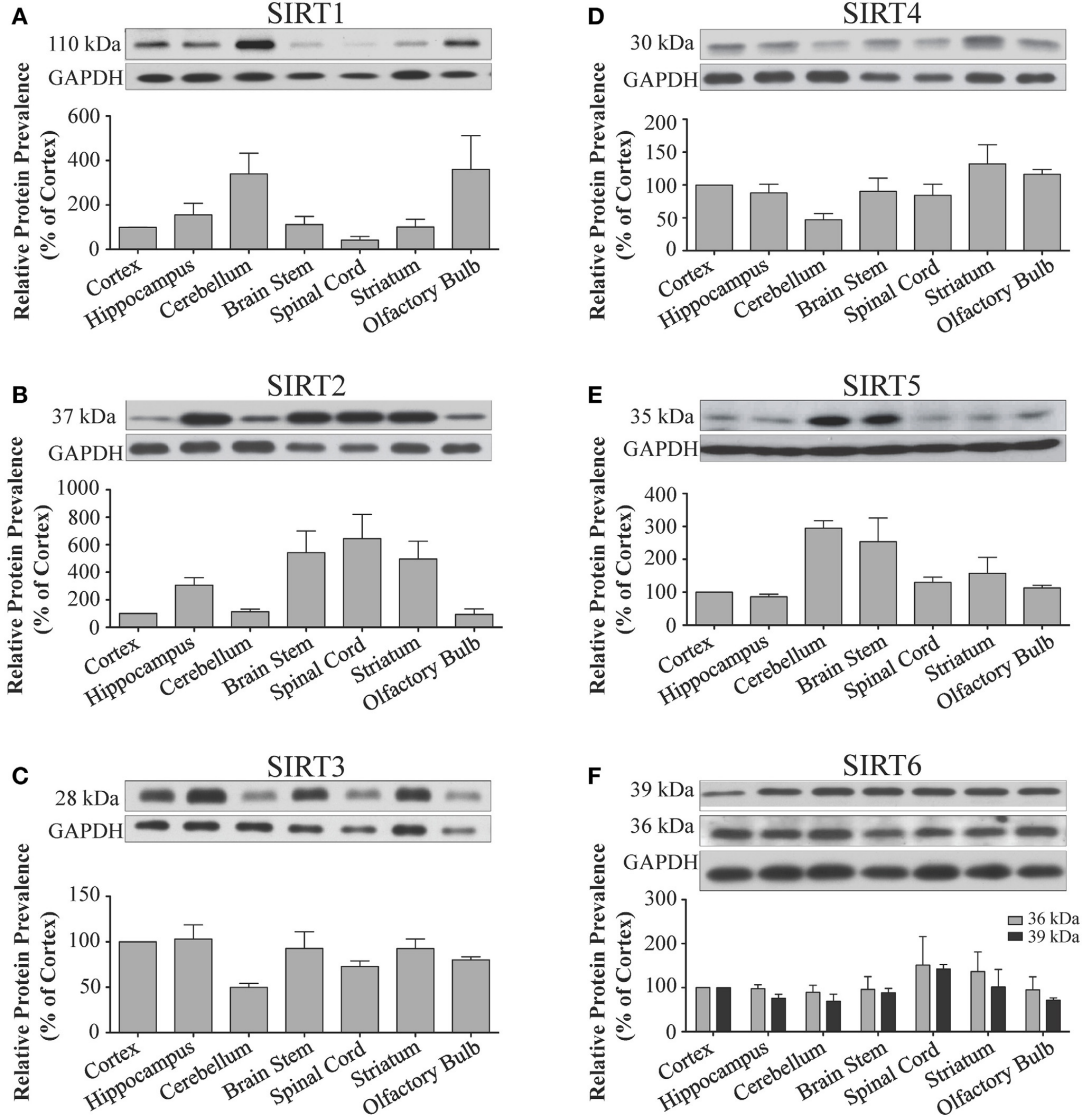
**Sirtuin protein expression patterns are non-redundant in different regions the adult rat brain**. Representative Western blots showing the immunoreactive protein levels for **(A)** SIRT1 **(B)** SIRT2 **(C)** SIRT3 **(D)** SIRT4 **(E)** SIRT5 **(F)** SIRT6 in the different regions of the adult rat brain as denoted. The histograms in each panel show the densitometric mean and SEM for each specific sirtuin normalized to its corresponding GAPDH loading control (*n* = 4 independent subjects). Statistical comparisons between sirtuin protein expression patterns in these adult brain regions are presented in Supplemental Table 3.

*Sirt1* mRNA was found by quantitative RT-PCR to be one of the least expressed sirtuin family members in brain. *Sirt1* mRNA levels were significantly lower than *Sirt2*, *Sirt3*, and *Sirt5* in the whole brain, and were present at similar levels to *Sirt4*, *Sirt6*, and *Sirt7* (Figure 1). Within different brain regions, *Sirt1* mRNA was found at similarly low levels, with only modest variability seen between different adult brain regions. The highest *Sirt1* mRNA levels were in cerebellum, while the lowest were in spinal cord (Figure 2A). At the protein level, siRNA treatment identified one specific SIRT1 product of 110 kDa (Supplemental Figure 1A). SIRT1 protein prevalence paralleled its mRNA pattern, being highest in the cerebellum, intermediate in the cortex, hippocampus, striatum and the olfactory bulb, and lowest in the spinal cord (Figure 3A). Relative to its expression in peripheral tissues, *Sirt1* mRNA levels in liver were similar to whole brain; spleen and kidney expressed higher levels of *Sirt1* mRNA than brain, while heart and small intestine expressed lower levels of *Sirt1* than brain (Table 1; Supplemental Figure 3A). This pattern was not recapitulated at the protein level, however, as SIRT1 protein expression was significantly higher in the brain than in any of these peripheral tissues (Supplemental Figure 4A).

**Table 1:** **Relative mRNA and protein expression patterns of specific sirtuin members in brain and peripheral tissues**.

**mRNA expression**	***Sirt1***	***Sirt2***	***Sirt3***	***Sirt4***	***Sirt5***	***Sirt6***	***Sirt7***
Whole brain	++	++++	++	+	+++	++	++
Heart	+	+++	++	+	+++	+	+
Liver	++	+++	++	+	++	+	++
Spleen	++	+++	++	+	++	+	++
Kidney	++	++++	++	++	+++	++	++
Intestine	+	+++	++	+	+++	++	+++
**Protein expression**	**SIRT1**	**SIRT2**	**SIRT3**	**SIRT4**	**SIRT5**	**SIRT6 (36 KDa)**	**SIRT6 (39 KDa)**
Whole brain	++	++++	+++	++	++	+	++
Heart	++	+++++	++++	++	++	+	++
Liver	++	+++++	+++	++	+++++	+	0
Spleen	0	+++++	0	+	+++	++	+
Kidney	0	+++	+++	++	+++++	+	+
Intestine	0	0	0	+	0	0	++

*In the upper mRNA expression panel, “+” are assigned based on the specified range of ΔCt values for the Sirtuin expression levels relative to the Hprt1 control gene, such as: +++++ = >3 ΔCt; ++++ = ΔCt 1.5 – 3; +++ = ΔCt 0.5 – 1.5; ++ = ΔCt 0.05 – 0.5; and += ΔCt < 0.05. In the lower protein expression panel, “+” are assigned based on their relative expression values for the sirtuin expression levels when compared to GAPDH control, such as: +++++, very high; ++++, high; +++, moderate; ++, low; and +, very low levels of expression. “0” was assigned to areas of no expression*.

**Figure 4:**
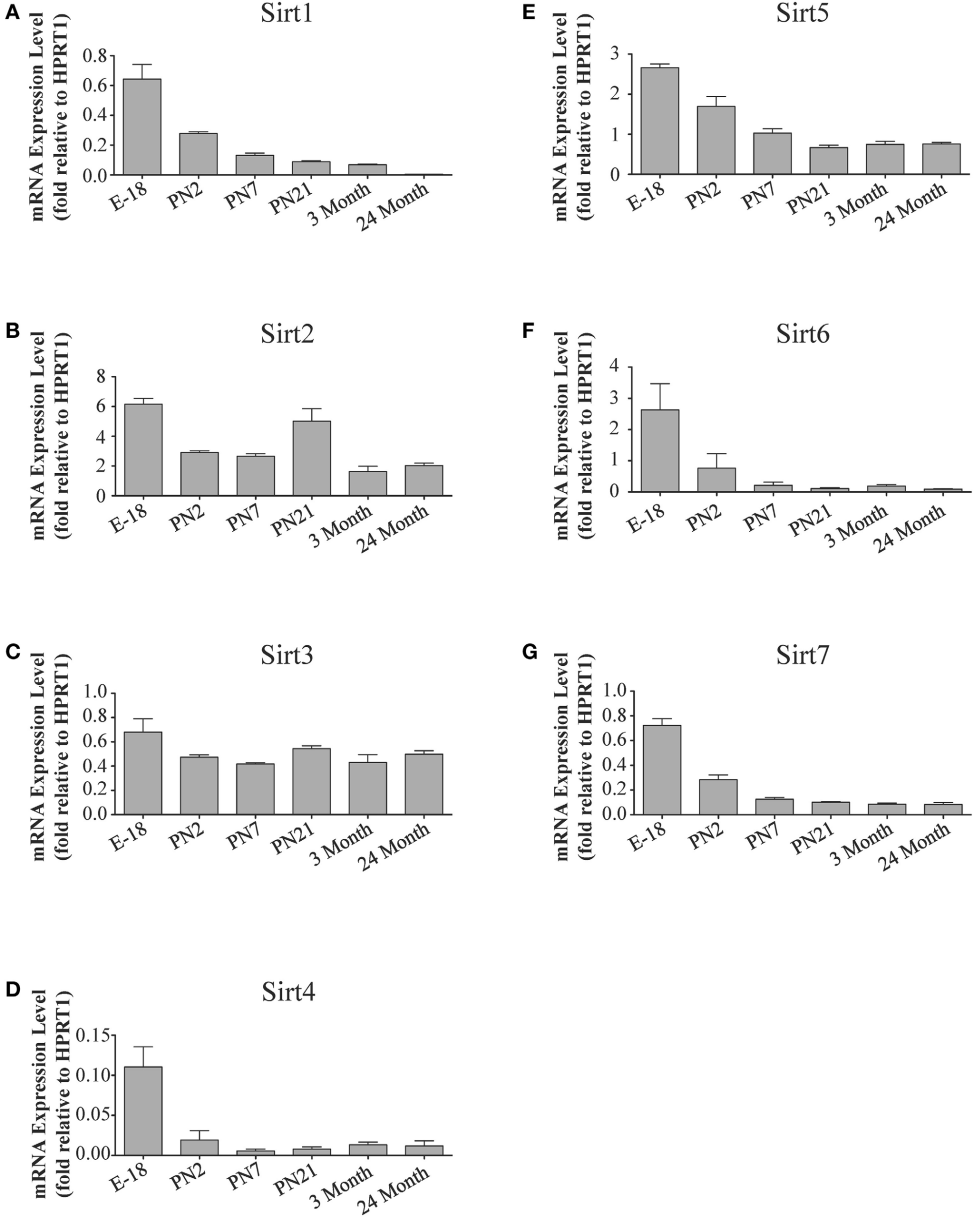
**Sirtuin mRNA expression patterns during rat cortical development**. Histograms showing the mean and SEM of the mRNA expression levels for each individual sirtuin at the indicated stages of cortical development (E18 to 3 months) and in rats at 24 months of age. The y-axis of each histogram shows the relative expression levels of **(A)** *Sirt1* **(B)** *Sirt2* **(C)** *Sirt3* **(D)** *Sirt4* **(E)** *Sirt5* **(F)** *Sirt6* **(G)** *Sirt7* relative to the levels of the *Hprt1* reference gene. Data shown are the linear conversion of delta CT values for each sample relative to *Hprt1* (*n* = 3 independent subjects done in quadruplicate). Statistical comparisons for the cortical expression levels of each sirtuin for the developmental times shown are presented in Supplemental Table 6.

**Figure 5:**
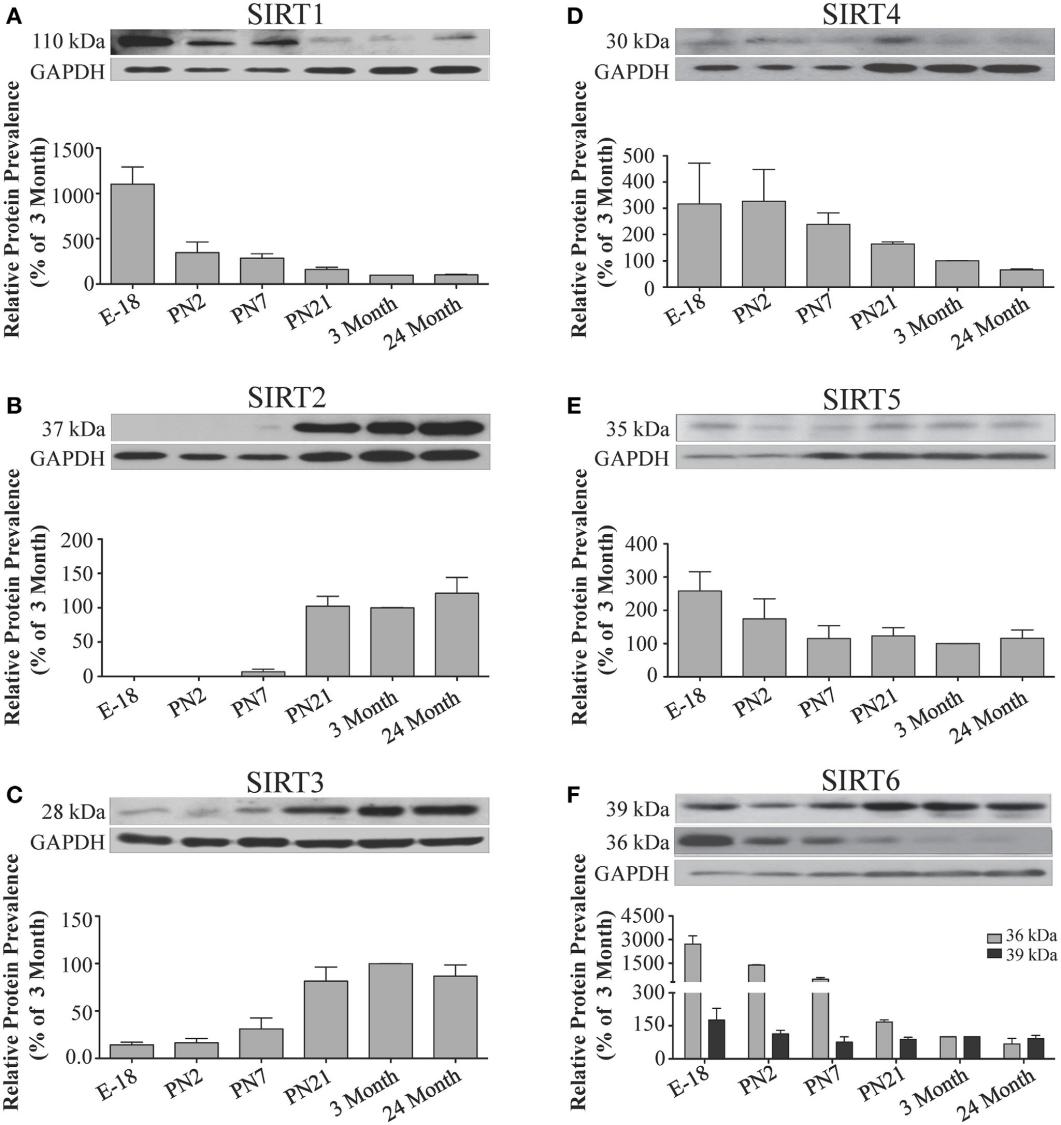
**Sirtuin protein expression patterns during rat cortical development**. Representative Western blots showing the prevalence of **(A)** SIRT1 **(B)** SIRT2 **(C)** SIRT3 **(D)** SIRT4 **(E)** SIRT5 **(F)** SIRT6 in the rat cortex at the indicated stages of embryonic and postnatal development (*n* = 3 independent subjects). The corresponding GAPDH protein level for the same blot is shown, and serves as a protein loading control. The histograms in each panel show the densitometric mean and SEM for each specific sirtuin normalized to its corresponding GAPDH loading control. The level of expression for each Sirtuin in the 3-month old cortex was arbitrarily set to 100%, and different stages expressed as a percentage of this 3-month value. Statistical comparisons for the expression levels of each sirtuin for the developmental times shown are presented in Supplemental Table 7.

**Figure 6:**
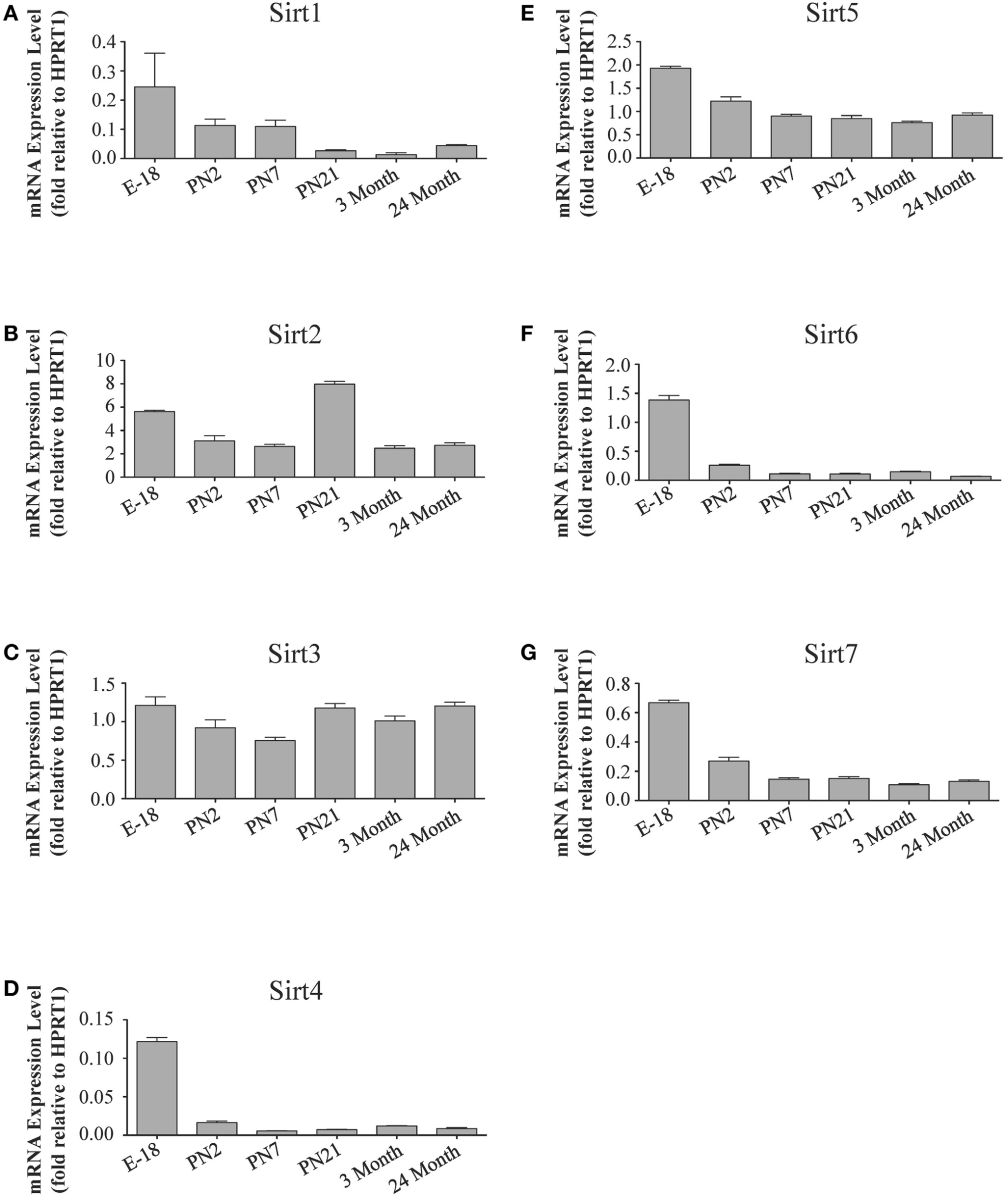
**Sirtuin mRNA expression patterns during rat hippocampal development**. Histograms showing the mean and SEM of the mRNA expression levels for individual sirtuins at the indicated ages and stage of embryonic hippocampal development. The y-axis of each histogram shows the expression levels of **(A)** *Sirt1* **(B)** *Sirt2* **(C)** *Sirt3* **(D)** *Sirt4* **(E)** *Sirt5* **(F)** *Sirt6* **(G)** *Sirt7* relative to the levels of the *Hprt1* reference gene. Data shown are based on the linear conversion of delta CT values from qRT-PCR assays for each sample (*n* = 3 independent subjects done in quadruplicate). Statistical comparisons for the hippocampal expression levels of each sirtuin for the developmental times shown are presented in Supplemental Table 8.

**Figure 7:**
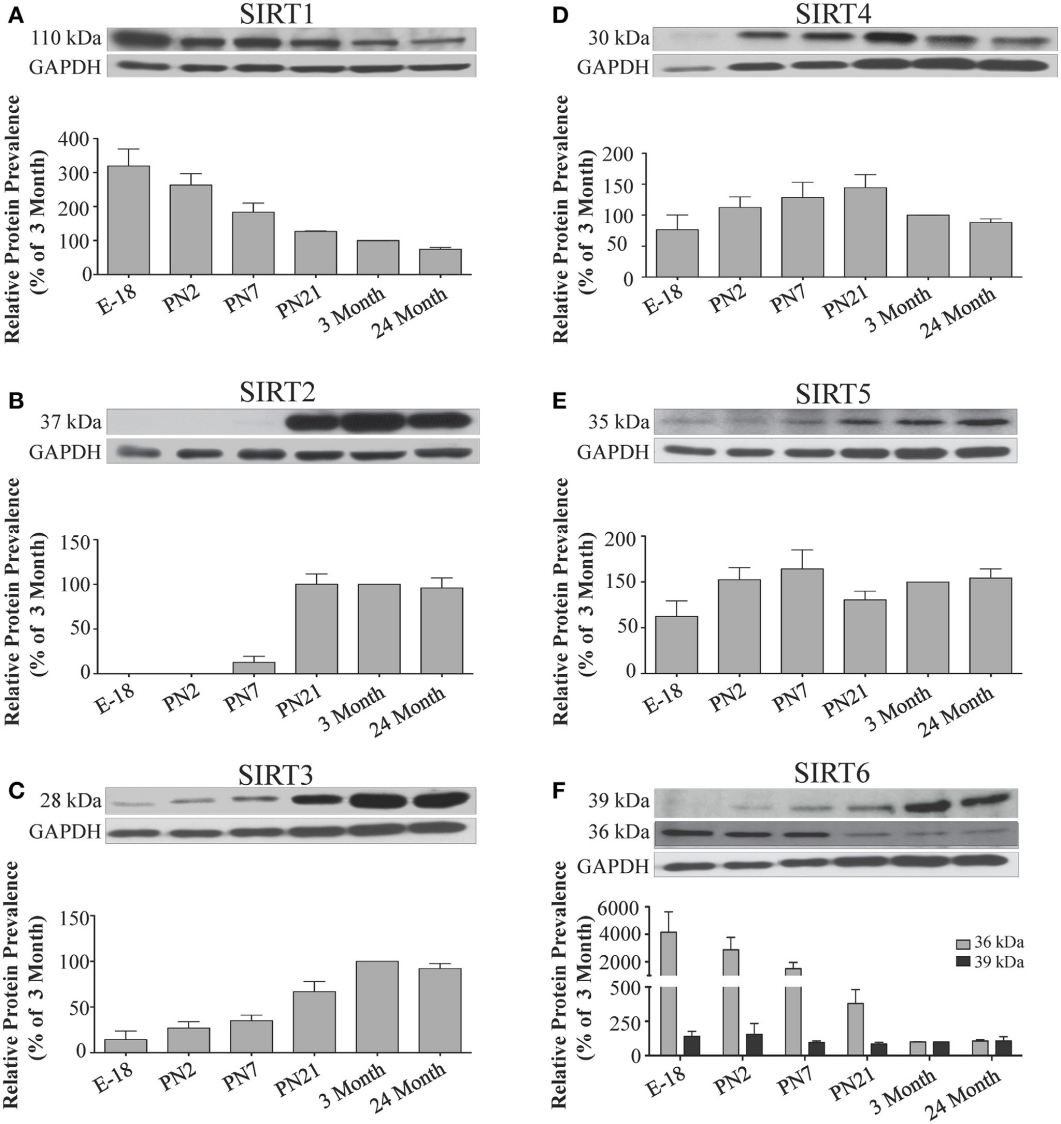
**Sirtuin protein expression patterns during rat hippocampal development**. Representative Western blots showing **(A)** SIRT1 **(B)** SIRT2 **(C)** SIRT3 **(D)** SIRT4 **(E)** SIRT5 **(F)** SIRT6 protein levels in rat hippocampus at the indicated embryonic or postnatal stages (*n* = 3 independent subjects). The corresponding GAPDH protein level for the same blot is shown, and serves as a protein loading control. The histograms show the densitometric mean and SEM for each specific sirtuin normalized to its corresponding GAPDH loading control, with the 3-month value arbitrarily set as 100%. Statistical comparisons for the expression levels of each sirtuin for the developmental times shown are presented in Supplemental Table 9.

**Figure 8:**
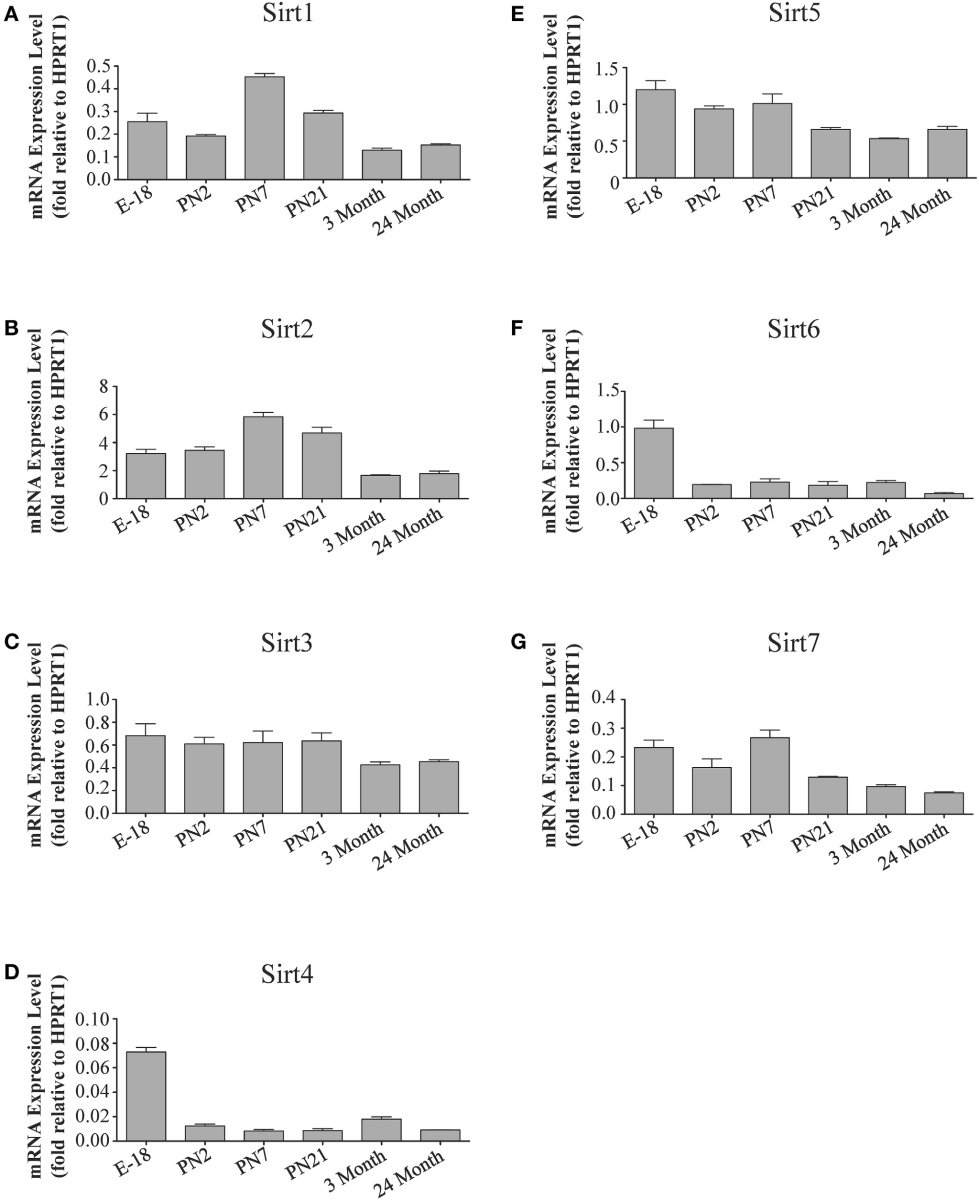
**Sirtuin mRNA expression patterns during rat cerebellum development**. Histograms showing the mean and SEM of the mRNA expression levels for individual sirtuins in the cerebellum at the indicated ages and stage of embryonic development. The y-axis of each histogram shows the expression levels of **(A)** *Sirt1* **(B)** *Sirt2* **(C)** *Sirt3* **(D)** *Sirt4* **(E)** *Sirt5* **(F)** *Sirt6* **(G)** *Sirt7* relative to the levels of the *Hprt1* reference gene. Data shown depict the linear conversion of delta CT values from qRT-PCR assays for each sample (*n* = 3 independent subjects done in quadruplicate). Statistical comparisons for the cerebellar expression levels of each sirtuin for the developmental times shown are presented in Supplemental Table 10.

**Figure 9:**
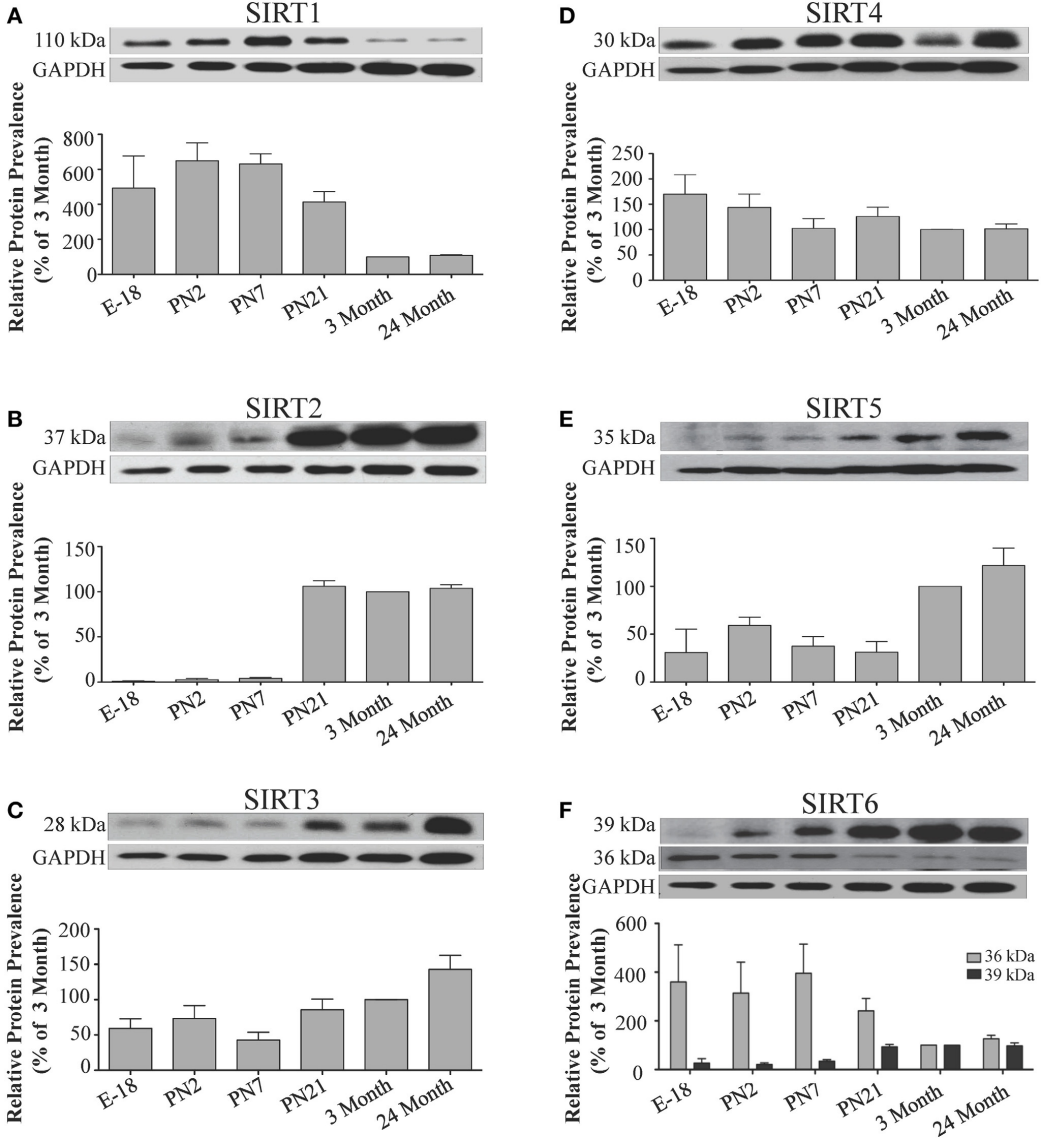
**Sirtuin protein expression patterns during rat cerebellum development**. Representative Western blots showing **(A)** SIRT1 **(B)** SIRT2 **(C)** SIRT3 **(D)** SIRT4 **(E)** SIRT5 **(F)** SIRT6 protein levels in rat cerebellum at the indicated embryonic or postnatal stages (*n* = 3 independent subjects). The corresponding GAPDH protein level for the same blot is shown, and serves as a protein loading control. The histograms show the densitometric mean and SEM for each specific sirtuin normalized to its corresponding GAPDH control, with the 3-month value arbitrarily set as 100%. Statistical comparisons for the expression levels of each sirtuin for the developmental times shown are presented in Supplemental Table 11.

During brain development, *Sirt1* mRNA levels decreased progressively relative to the reference gene *Hprt1* from the late embryonic stage in cortex, hippocampus, and cerebellum (Table 2; Figures 4A, **6A**, **8A**). This developmental mRNA expression pattern was not brain-specific, as a similar pattern was also observed in liver (Supplemental Figure 5A; Supplemental Table 12). At the protein level, this general pattern was recapitulated, as SIRT1 protein expression was significantly higher in cortex and hippocampus at E18 and early postnatal stages compared to the adult and 24 month old stages (Table 2; Figures 5A, **7A**). A slightly different pattern was observed in cerebellum, as while SIRT1 protein levels were lower in adult than in perinatal stages, the decrease in prevalence did not occur until after postnatal day 21 (**Figure 9A**). In each brain region, SIRT1 protein prevalence at 3 and 24 months were similar, respectively, indicating the absence of an aging-associated decrease in SIRT1 protein prevalence. This progressive decrease in SIRT1 protein during perinatal development appeared to be brain-selective, as SIRT1 protein levels increased in the liver during perinatal development, and then remained at similar levels until the 24 month stage (Supplemental Figure 6A; Supplemental Table 13).

**Table 2:** **Developmental mRNA and protein expression patterns of sirtuin members in cortex, hippocampus, and cerebellum**.

**mRNA expression**	***Sirt1***			***Sirt2***			***Sirt3***			***Sirt4***			***Sirt5***			***Sirt6***			***Sirt7***		
	**Cor**	**Hip**	**Cer**	**Cor**	**Hip**	**Cer**	**Cor**	**Hip**	**Cer**	**Cor**	**Hip**	**Cer**	**Cor**	**Hip**	**Cer**	**Cor**	**Hip**	**Cer**	**Cor**	**Hip**	**Cer**
E-18	+	+	+	+++++	+++++	+++++	+++	+++	+++	++	++	++	++++	++++	+++	++++	+++	+++	+++	+++	++
PN2	+	+	+	++++	++++	+++++	++	+++	+++	+	+	+	++++	+++	+++	+++	++	++	++	++	++
PN7	+	+	+	++++	++++	+++++	++	+++	+++	+	+	+	+++	+++	+++	++	++	++	++	++	++
PN21	+	+	+	++++	++++	+++++	++	+++	+++	+	+	+	+++	+++	+++	++	++	++	++	++	++
3 months	+	+	+	++++	++++	++++	++	+++	++	+	+	+	+++	+++	+++	++	++	++	++	++	++
24 months	+	+	+	++++	++++	++++	++	+++	++	+	+	+	+++	+++	+++	++	++	++	++	++	++
**Protein expression**	**SIRT1**			**SIRT2**			**SIRT3**			**SIRT4**			**SIRT5**			**SIRT6 (36 kDa)**			**SIRT6 (39 kDa)**		
	**Cor**	**Hip**	**Cer**	**Cor**	**Hip**	**Cer**	**Cor**	**Hip**	**Cer**	**Cor**	**Hip**	**Cer**	**Cor**	**Hip**	**Cer**	**Cor**	**Hip**	**Cer**	**Cor**	**Hip**	**Cer**
E-18	+++	+++	+++	0	0	+	+	+	+	+	+	+	++	++	++	++++	+++	++	+	+	+
PN2	+++	+++	+++	0	0	+	+	+	+	+	+	+	++	++	++	+++	+++	++	+	+	+
PN7	+++	+++	+++	0	0	+	+	+	+	+	+	+	++	++	++	+++	+++	++	+	+	+
PN21	+++	++	+++	++++	+++++	++++	+++	+++	++	+	+	+	++	++	++	+	+	+	+	+	+
3 months	++	++	+	++++	+++++	++++	+++	+++	++	+	+	+	++	++	++	+	+	+	+	+	+
24 months	++	+	+	++++	+++++	++++	+++	+++	+++	+	+	+	++	++	++	+	+	+	+	+	+

*In the upper mRNA expression panel, “+” are assigned based on the specified range of ΔCt values for the Sirtuin expression levels relative to the Hprt1 control gene, such as: +++++= >3 ΔCt; ++++ = ΔCt 1.5 – 3; +++ = ΔCt 0.5 – 1.5; ++ = ΔCt 0.05 – 0.5; and += ΔCt < 0.05. In the lower protein expression panel, “+” are assigned based on their relative expression values for the sirtuin expression levels when compared to GAPDH control, such as: +++++, very high; ++++, high; +++, moderate; ++, low; and +, very low levels of expression. “0” was assigned to areas of no expression*.

**Figure 10:**
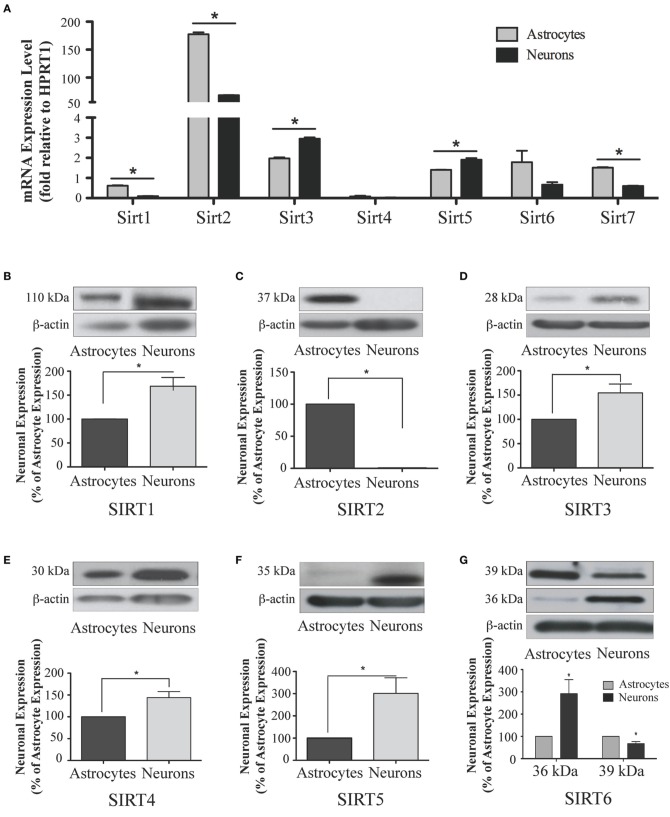
**Sirtuin mRNA expression in mature rat astrocytes and neurons. (A)** Histogram showing the mean and SEM of *Sirt1-7* mRNA expression levels in primary cultures of astrocytes (gray bars) and neurons (black bars). The y-axis shows the relative mRNA expression levels relative to the endogenous housekeeping *Hprt1* reference gene in each culture condition. Data shown depict the linear conversion of delta CT values from qRT-PCR assays for each sample (*n* = 3 independent subjects done in quadruplicate). Asterisks denote significant differences in the relative expression of the specific sirtuin between astrocyte and neuronal cultures (*p* < 0.05, Student's *t*-test). **(B–G)** Representative Western blots showing the relative immunoreactive levels of **(B)** SIRT1, **(C)** SIRT2 **(D)** SIRT3 **(E)** SIRT4 **(F)** SIRT5 **(G)** SIRT6 in cultured astrocytes and neurons. The histograms show the mean and SEM of the densitometric band intensity for each sirtuin normalized to its corresponding β-actin levels. Asterisks denote significant differences in expression levels for the indicated sirtuin between the neuronal and astrocyte cultures (*n* = 3, *p* < 0.05, student's *t*-test).

Analysis of *Sirt1* expression between astrocytes and neurons revealed that while *Sirt1* mRNA was detected in extracts from each of these respective cultures, its mRNA levels were 84.0 ± 0.3% higher in the astrocyte cultures than in the neuronal cultures (*p* < 0.05; **Figure 10A**). Consistent with its low expression in the adult brain, *Sirt1* mRNA was also relatively low compared to the other sirtuins except *Sirt4* in each of these cultured cell types. At the protein level, though, SIRT1 prevalence differed somewhat from its mRNA profile in these cultures. Although SIRT1 protein was also detected in both the neuron and astrocyte-enriched cultures, its overall prevalence was 68.5 ± 18.4% higher in the neuronal cultures than in the astrocytes (*p* < 0.05; **Figure 10B**).

### Sirt2

Quantitative RT-PCR revealed that *Sirt2* is the most abundantly expressed sirtuin in the rat brain at the mRNA level (Figure 1), where its expression was found to be more than 3-fold higher than the reference *Hprt1* gene (Supplemental Figure 2B). Within the different adult brain regions, *Sirt2* mRNA was highest in spinal cord, brain stem, and striatum, and at similar levels in cortex, hippocampus, cerebellum and olfactory bulb (Figure 2B). At the protein level, SIRT2 protein expression patterns were similar its mRNA patterns, being most pronounced in the spinal cord and brain stem, but still showing strong immunoreactive prevalence in cortex, hippocampus, striatum and cerebellum (Figure 3B). *Sirt2* mRNA was also present at high levels relative to its family members in peripheral tissues, but only the kidney expressed *Sirt2* mRNA at levels equivalent to those in the adult brain (Table 1; Supplemental Figure 3B). At the protein level, siRNA treatment identified one specific SIRT2 protein product of 37 kDa (Supplemental Figure 1B). SIRT2 protein prevalence in heart, liver, and spleen were similar to its brain levels, while its protein prevalence in small intestine and kidney were at lower levels than in the adult brain (Table 1; Supplemental Figure 4B).

The developmental expression pattern of *Sirt2* mRNA relative to the *Hprt1* reference gene in the cortex and hippocampus was highest at E18, and then remained fairly consistent across perinatal development and into adulthood (Table 2; Figures 4B, 6B). A spike of *Sirt2* mRNA was observed at PN21, but this increase was transient and not retained in the 3 month old cortex or hippocampus (Figures 4B, 6B). The expression profile of *Sirt2* was somewhat different in the cerebellum, where *Sirt2* mRNA displayed a transient increase at PN7 and PN21 compared to E18 and PN2, and then decreased to consistent levels seen in both the adult and 24 month old tissues (**Figure 8B**). Intriguingly, however, this mRNA profile for *Sirt2* was not recapitulated at the protein level, as SIRT2 immunoreactivity was minimal or at background levels in samples prior to the PN21 stage in cortex, hippocampus, and cerebellum. SIRT2 protein then rapidly appeared at PN21 in each of these brain regions, and remained at fairly consistent levels until 24 months of age (Table 2; Figures 5B, 7B, **9B**). In adult peripheral tissues, *Sirt2* mRNA and protein were expressed at varying levels relative to brain, with SIRT2 protein in heart, liver and spleen similar to or slightly above those found in adult brain (Supplemental Figures 3B, 4B). During liver development, *Sirt2* mRNA was expressed from PN2-24 months at similar levels, but was modestly higher at the E18 stage (Supplemental Figure 5B; Supplemental Table 12). SIRT2 protein in the developing liver largely paralleled this pattern, but unlike *Sirt2* mRNA, SIRT2 protein displayed a significant increase in prevalence at the 3 month stage, before returning to levels similar to perinatal times at 24 months of age (Supplemental Figure 6B; Supplemental Table 13).

The analysis of *Sirt2* expression in the neuron and astrocyte cultures also yielded intriguing results. Consistent with its expression levels in whole brain, *Sirt2* mRNA was the most prominently expressed sirtuin family member in each culture, although the astrocytes displayed approximately 7-fold higher Sirt2 mRNA than neurons (**Figure 10A**). This mRNA pattern was not recapitulated at the protein level, however. In fact, while SIRT2 immunoreactivity was readily detected in extracts from the astrocyte-enriched cultures, its immunoreactivity in extracts from the neuronal cultures was at, or only slightly above, background levels (**Figure 10C**). Thus, despite having pronounced mRNA expression in neurons, SIRT2 protein was not readily detected in the same cultured neurons.

### Sirt3

At the mRNA level, *Sirt3* was the third most expressed member of the sirtuin family in the whole brain (Figure 1), and in each of the different adult brain regions examined except cerebellum, where its expression was modestly lower (Figure 2C). At the protein level, SIRT3 specific products were identified using cortical tissue derived from a SIRT3-null mouse (Lombard et al., 2007), which identified one specific SIRT3 product of 28 kDa (Supplemental Figure 1C). SIRT3 protein levels largely paralleled its mRNA expression patterns in the adult brain, being at similar levels within cortex, hippocampus, striatum, spinal cord and brain stem, and at modestly lower levels in the cerebellum (Figure 3C). With respect to peripheral tissues, the kidney displayed *Sirt3* mRNA levels similar to those of whole brain, while each of the other tissues examined showed lower *Sirt3* mRNA levels than brain (Table 1; Supplemental Figure 3C). At the protein level, though, there was considerable variations in SIRT3 immunoreactivity in these tissues: SIRT3 protein prevalence in heart was higher than in brain, the spleen and small intestine had lower SIRT3 expression than brain, and the kidney and liver had similar SIRT3 levels as brain (Table 1; Supplemental Figure 4C).

The mRNA expression pattern of *Sirt3* during development was relatively similar in cortex, hippocampus, and cerebellum, with *Sirt3* mRNA levels being fairly consistent from E18 until 24 months of age (Table 2; Figures 4C, 6C, 8C). The pattern of SIRT3 protein expression was quite different, however, as a robust increase in SIRT3 protein prevalence was observed in cortex and hippocampus between PN7 and PN21, and this level was maintained in the 3 and 24 month old samples (Table 2; Figures 5C, 7C). In cerebellum, SIRT3 protein prevalence was similar between E18 to 3 months, but then increased significantly at 24 months of age (Figure 9C). *Sirt3* mRNA and protein expression in the liver across development displayed similarities to its brain expression pattern, as *Sirt3* mRNA and protein levels increased dramatically between E18 to PN7, then decreased by PN21 to levels that remained similar at 3 and 24 months of age (Supplemental Figures 5C, 6C; Supplemental Tables 12 and 13).

Within the neuronal and astrocytes cultures, while *Sirt3* mRNA was present in both cell types, *Sirt3* mRNA levels were 33.1 ± 0.3% higher in the neuronal cultures than in the astrocyte cultures (*p* < 0.05; Figure 10A). This pattern held at the protein level also, as SIRT3 protein was 63.5 ± 12.7% more prevalent in extracts from the neuronal cultures compared the astrocyte cultures (Figure 10D).

### Sirt4

*Sirt4* was found by quantitative RT-PCR to be the least abundantly expressed member of the sirtuin family in the adult rat brain (Figure 1). Its mRNA expression levels ranged from 0.5 to 2% of the reference gene *Hprt1* throughout the different regions of the brain, except for spinal cord, which had the highest level of *Sirt4* expression at 5% of *Hprt1* (Figure 2D). This low level of expression was also seen in the peripheral tissues, where only the kidney expressed *Sirt4* at higher levels than brain (Table 1; Supplemental Figure 3D). At the protein level, siRNA treatment identified one specific SIRT4 protein product with a mass of 30 kDa (Supplemental Figure 1D). SIRT4 protein expression was detected in each of the brain regions examined at relatively similar levels (Figure 3D). This general pattern was also seen in the peripheral tissues, where SIRT4 protein prevalence in heart, liver, and kidney were at levels similar to that of brain, while the spleen and small intestine displayed lower levels of SIRT4 protein than adult brain (Table 1; Supplemental Figure 4D).

During development, *Sirt4* mRNA levels in each of the brain regions examined were highest at the E18 stage, decreased significantly from this late embryonic level by PN2, and then remained at fairly consistent levels between PN7 and 24 months of age (Table 2; Figures 4D, 6D, 8D). At the protein level, no significant differences in SIRT4 protein prevalence were noted in any of the brain regions examined throughout development, including the E18 time point where higher mRNA levels had been seen (Table 2; Figures 5D, 7D, 9D). A similar pattern of mostly consistent expression was also observed in the developing liver, as *Sirt4* mRNA levels decreased modestly between E18 to and PN2, and then remained fairly stable until 24 months of age (Supplemental Figure 5D; Supplemental Table 12). Similarly, SIRT4 protein levels did not significantly differ in liver from E18 to 24 months of age (Supplemental Figure 6D; Supplemental Table 13).

Consistent with its low levels of expression levels in the brain and peripheral tissues, *Sirt4* mRNA was also the least prevalently expressed sirtuins member in both the neuronal and astrocyte enriched cultures, and no significant difference in *Sirt4* mRNA prevalence was observed between the neurons and astrocytes (Figure 10A). At the protein level, SIRT4 protein expression was also detected in each cell type, although SIRT4 protein was 41.2 ± 0.2% higher in the neuronal cultures compared to astrocytes (*p* < 0.05; Figure 10E).

### Sirt5

Quantitative RT-PCR revealed that *Sirt5* mRNA is expressed at levels similar to *Hprt1* in whole brain homogenates, making it the second most prevalently expressed member of the sirtuin family in the adult brain (Figure 1). However, differences in the magnitude of *Sirt5* expression were observed between different regions of the adult brain. *Sirt5* mRNA was significantly higher in brain stem, hippocampus, spinal cord, and olfactory bulb than in cortex and cerebellum (Figure 2E). At the protein level, siRNA treatment identified one specific SIRT5 product of 35 kDa (Supplemental Figure 1E). The adult mRNA expression profile was generally recapitulated at the protein level, although SIRT5 protein in cerebellum was higher than in cortex and hippocampus (Figure 3E). In peripheral tissues, *Sirt5* mRNA expression in kidney was at levels similar to brain, while all of the other peripheral tissues examined expressed *Sirt5* mRNA at levels lower than brain (Supplemental Figure 3E). The protein profile of SIRT5 in these peripheral tissues did not parallel its mRNA pattern, however, as SIRT5 protein was notably higher in liver, spleen, and kidney than in whole brain, and present at lower or non-detectable levels in small intestine and heart (Table 1; Supplemental Figure 4E).

During development, *Sirt5* mRNA levels decreased modestly in the cortex and hippocampus between E18 and PN7, and then remained at consistent levels until 24 months of age (Table 2; Figures 4E, 6E). In the developing cerebellum, *Sirt5* mRNA levels decreased at 3 months of age, and this lower level remained in tissue from 24 month old rats (Figure 8E). At the protein level, SIRT5 immunoreactivity did not significantly differ across development within either the cortex or hippocampus (Figures 5E, 7E), although SIRT5 protein levels in the cerebellum were higher at 3 and 24 months when compared to levels seen at E18 or in the perinatal stages (Table 2; Figure 9E). During liver development, *Sirt5* mRNA expression decreased between E18 and PN2, and then remained fairly consistent until 24 months of age (Supplemental Figure 5E; Supplemental Table 12). The pattern of SIRT5 protein differed from this mRNA profile in liver, however, as SIRT5 protein levels were found to increase progressively until PN21, and then remained at similar levels at 3 and 24 months of age (Supplemental Figure 6E; Supplemental Table 13).

*Sirt5* mRNA was present at relatively high levels relative to other Sirtuins in both the neuronal and astrocytic cultures, with its mRNA expression being moderately but significantly higher in neurons by 26.4 ± 0.3% than in astrocytes (Figure 10A). At the protein level, SIRT5 prevalence was more skewed in its neuronal preference, being 201.6 ± 60.8% higher in the neuronal cultures than in the astrocyte cultures (*p* < 0.05; Figure 10F).

### Sirt6

*Sirt6* mRNA was found at relatively low levels in the brain by quantitative RT-PCR, being expressed at approximately 10% the level of the *Hprt1* reference gene (Figure 1). The highest mRNA expression for *Sirt6* was observed in the olfactory bulb, and the lowest expression levels were found in hippocampus and brain stem (Figure 2F). A similar low level of mRNA expression was also seen in peripheral tissues, with only the kidney displaying higher *Sirt6* mRNA levels than brain, and the liver and heart showing lower expression than brain (Table 1; Supplemental Figure 3F). At the protein level, siRNA treatment revealed two specific SIRT6 protein products with masses of 36 kDa and 39 kDa (Supplemental Figure 1F). SIRT6 protein expression for each of the isoforms was similar within the different adult brain regions examined (Figure 3F). In adult peripheral tissues, the expression levels of these two SIRT6 protein forms was variable, with the 36 kDa being expressed highest in the spleen, where its prevalence was greater than in brain, whereas the intestine expressed low or non-detectable levels of this 36 kDa form (Table 1; Supplemental Figure 4F). In contrast, the 39 kDa form was expressed at levels similar to brain in heart and intestine, while liver displayed low to non-detectable levels of this 39 kDa SIRT6 form.

In the developing brain, *Sirt6* mRNA levels were highest in the cortex, hippocampus, and cerebellum at the E18 stage, and then decreased to levels at PN7 that were maintained at 24 months (Table 2; Figures 4F, 6F, 8F). *Sirt6* mRNA expression in the developing liver similarly decreased during early perinatal development from its late embryonic level, and remained at similar levels until 24 months of age (Supplemental Figure 5F; Supplemental Table 12). At the protein level, the different forms of expressed SIRT6 protein displayed region-specific differences in expression. Throughout cortical and hippocampal development, the prevalence of the 36 kDa product decreased from E18 to PN21, and then remained at similar levels in these respective tissues at 3 and 24 months of age. This was not the case for the 39 kDa SIRT6 protein product, though, as its prevalence remained constant throughout cortical and hippocampal development (Table 2; Figures 5F, 7F). In the developing cerebellum, the 36 kDa SIRT6 product was found at similar levels throughout development, while the 39 kDa form increased in prevalence between E18 and PN7, and then remained at fairly consistent levels until 24 months of age (Table 2; Figure 9F). In the developing liver, the 36 kDa SIRT6 protein form was found at consistent levels throughout development, whereas the prevalence of the 39 kDa product was at low or background levels for each of the developmental stages examined (Supplemental Figure 6F; Supplemental Table 13).

In the neuronal and astrocyte-enriched culture extracts, *Sirt6* mRNA was detected in both cell lineages, and at similar levels (Figure 10A). At the protein level, SIRT6 immunoreactivity was also found in both the neuronal and astrocyte-enriched cultures, although both the 36 kDa and the 39 kDa protein products displayed preferential expression in one cell type. The prevalence of the 36 kDa SIRT6 product was 191.6 ± 63.8% higher in the neuronal cultures than in the astrocytes (*p* < 0.05; Figure 10G), while the prevalence of the 39 kDa SIRT6 protein form was 32.8 ± 9.5% higher in the astrocyte cultures compared to the neurons (*p* < 0.05; Figure 10G).

### Sirt7

Quantitative RT-PCR revealed that *Sirt7* is also expressed at low levels in the adult brain relative to other sirtuin family members, being present at roughly 5% the levels of the *Hprt1* reference gene (Figure 1). Within the different regions of the adult brain examined, no significant differences in *Sirt7* mRNA expression was detected (Figure 2G). Interestingly, and in contrast to the other sirtuin family members, *Sirt7* mRNA expression was highest in the small intestine (Table 1; Supplemental Figure 3G), where its expression levels were about 10-fold higher than its mRNA levels in brain. Although lower than in small intestine, both the spleen and kidney also showed significantly higher *Sirt7* mRNA expression levels than the brain.

During cortical and hippocampal development, *Sirt7* mRNA levels decreased between E18 and PN2, but then remained at similar levels throughout the remaining time points examined (Table 2; Figures 4G, 6G). In the cerebellum, *Sirt7* mRNA levels remained at relatively similar levels throughout perinatal development, as well as in the adult and 24 month old brain (Table 2; Figure 8G). Across liver development, *Sirt7* mRNA levels decreased modestly from E18 to PN21, and then remained at similar levels at 3 and 24 months (Supplemental Figure 5G; Supplemental Table 12). In the neuronal and astrocyte-enriched culture extracts, *Sirt7* mRNA was detected in both lineages, with its expression in astrocytes being 60.1 ± 0.1% higher than in neuronal cultures than in astrocytes (*p* < 0.05; Figure 10A). SIRT7 protein expression patterns could not be determined in this study, as we failed to identify a SIRT7 antibody that yielded a product that could be suppressed by *Sirt7* targeted siRNA treatment (see Materials and Methods and Supplemental Figure 1G).

## Discussion

In this study we assessed the expression patterns of seven sirtuin family members in specific regions of the developing, adult, and aged rat brain (Table 2). *Four* principle findings emerge from our study. First, although each of the sirtuin family members is expressed within the adult and developing rat brain, there is considerable variability in the magnitude of mRNA expression between each of the individual sirtuins, with *Sirt2* being the most prominently expressed and *Sirt4* being the least expressed. Second, although there is some overlap between specific members, individual sirtuin family members display a unique pattern of temporal and spatial expression within the adult and developing brain. Third, specific members of the sirtuin family display preferential or even selective cell type expression patterns between neurons and astrocytes at the mRNA and protein levels. Fourth, there is notable discordance between the mRNA and protein expression patterns for Sirt2, indicating that its protein expression pattern is likely regulated by post-transcriptional mechanisms. Collectively, these results reveal a contrasting expression pattern for the seven sirtuin family members in the developing and adult brain, and suggest that metabolic processes and/or oxidative defense systems within different regions of the brain, and potentially within specific neural cell types, may be differentially regulated by specific combinations of sirtuins.

Although previous studies have indicated different sirtuins are expressed within the brain (Ford et al., 2006; Mostoslavsky et al., 2006; Li et al., 2007; Lombard et al., 2007; Ramadori et al., 2008; Nakagawa et al., 2009), our results indicate that there is considerable divergence amongst the family members in their spatial expression patterns, in their developmental profile of expression, and in the magnitude of their mRNA abundance within specific brain regions. Although none of the adult brain regions we examined was devoid of expressing any sirtuin member, the three most prevalently expressed sirtuins within each brain region were *Sirt2*, *Sirt3*, and *Sirt5*. This general pattern was also recapitulated in the peripheral tissues we examined, although in some peripheral tissues *Sirt7* mRNA was more prevalent that *Sirt5* or *Sirt3*. In fact, *Sirt7* mRNA levels were significantly higher in peripheral tissues than *Sirt7* mRNA levels in any region of the adult brain we tested. This differential *Sirt7* mRNA expression pattern may indicate a more prominent role for *Sirt7* in tissues with high cell proliferation rates as suggested previously (Ford et al., 2006), as compared to its role within the largely post-mitotic brain.

During development, *Sirt1*, *Sirt6*, and *Sirt7* mRNA levels were significantly higher in the late embryonic and early postnatal regions of the brain than in the corresponding adult brain regions. Only *Sirt4* displayed lower mRNA levels than *Sirt1*, *Sirt6*, and *Sirt7* in these brain regions during early perinatal development or in the adult stage. The relatively low mRNA levels observed for *Sirt1*, as well as for *Sirt6* and *Sirt7*, in brain regions from 3 and 24 month old rats relative to their expression levels in the late embryonic and early postnatal times were somewhat surprising, as *Sirt1* has been strongly implicated as a neuroprotective factor in models of Huntington's disease (Jeong et al., 2011), Multiple sclerosis (Shindler et al., 2007; Khan et al., 2014), stroke (Yan et al., 2013) and Alzheimer's disease (Qin et al., 2006). However, low basal expression levels could potentially be advantageous for these sirtuins in the brain, as SIRT1 levels have been shown to increase significantly in different cultured cell lines when they are exposed to conditions of energy/nutrient stress (Kanfi et al., 2008; Chen et al., 2011; Li et al., 2013). Similarly, SIRT6 and SIRT7 have been implicated in the maintenance of DNA integrity, and in DNA repair following damage (Ford et al., 2006; Cardus et al., 2013), which is often a consequence of increased cellular stress. Therefore, the relatively lower levels of *Sirt1*, *Sirt6*, and *Sirt7* present in the adult brain under normal conditions may allow for their pronounced induction under pathophysiological conditions to facilitate a beneficial compensatory response in times of need. It is worth noting that neither the mRNA levels or protein prevalence of either SIRT1 or SIRT6 in the 24 month old cortex or hippocampus differed from their respective levels at 3 months of age, suggesting that aging alone does not alter the expression of these sirtuins. While this suggests the lack of an endogenous age-related compensatory response to aging, these results do confirm the expression of each sirtuin in the aged brain, which therefore validates their pharmacological targeting for age-related conditions. Indeed, brain-specific SIRT1 over-expressing transgenic mice show significant life span extensions (Satoh et al., 2013), suggesting enhancing SIRT1 activity pharmacologically in the brain may yield a similar effect. Likewise, the global over-expression of SIRT6 in transgenic mice similarly extended life span (Kanfi et al., 2012), suggesting SIRT6 may also be a rationale target for drug development. In our study, analysis of SIRT6 protein expression revealed two products at 36 kDa and 39 kDa, which comports with a recent study examining how preterm labor affects SIRT6 expression that also reported the presence of two distinct SIRT6 products (Lim et al., 2013). Interestingly, we show that these distinct SIRT6 forms displayed somewhat opposing expression patterns during brain development: while the 36 kDa product decreased during perinatal development, the 39 kDa product increased as the animal aged. While the reason for the divergent expression pattern remains unknown, these results indicate that an examination of the role played by each in temporal brain development and aging is warranted.

Analysis of the astrocytic and neuronal expression patterns of the predominantly nuclear residing Sirt1, Sirt6, and Sirt7 sirtuins revealed that while each sirtuin is expressed in these two neural cell lineages, their relative expression levels are not equivalent between the two cell types. At the mRNA level, *Sirt1*, *Sirt6*, and *Sirt7* displayed modestly (approximately 2-fold) higher expression in astrocytes than in neurons relative to the reference gene *Hprt1*. At the protein level, this pattern of more predominant astrocyte expression was not recapitulated by SIRT1, whose protein prevalence was found to be significantly higher in neurons than in astrocytes. This differential expression profile of Sirt1 mRNA and protein between cultured astrocytes and neurons suggests that post-transcriptional factors may play a role in establishing SIRT1 protein expression patterns in these specific cell types. Importantly, these results also indicate that glial cells should be considered as potential mediators of neuroprotective actions associated with SIRT1-activating drugs (Shindler et al., 2007; Khan et al., 2014). Somewhat surprisingly, SIRT6 protein expression was found to display isoform-specific preferences between neurons and astrocytes. This could indicate the two forms influence specific cell types in the brain in different or specific manners. Favero et al. (2014) recently reported SIRT6 immunoreactivity within the nucleus of cortical neurons in mouse brain. Although this study did not resolve specific forms of SIRT6, or explicitly examine SIRT6 expression in astrocytes, the presence of SIRT6 immunoreactivity in neurons is consistent with our results. In this regard, it is of interest to note that the selective ablation of SIRT6 from neurons in mice results in an adult-onset obesity (Schwer et al., 2010), while liver-specific deletion of *Sirt6* in mice results in a “fatty liver” phenotype (Kim et al., 2010). Our results indicate that the 36 kDa product is the form most predominately expressed in neurons, and is the only form reliably detected in the developing and adult liver. By extrapolation, this would suggest the loss of the 36 kDa form may be predominantly responsible for the observed phenotypes in these respective studies. At the least, our results confirm that both SIRT1 and SIRT6 proteins are expressed in varying levels in both neurons and astrocytes which therefore indicates drugs designed to enhance the catalytic activity of either SIRT1 or SIRT6 should elicit effects within both of these cell types—but potentially with differing cell-selective efficacies depending on the sirtuin, or the SIRT6 form, that is being pharmacologically targeted.

In contrast to the relative low expression levels of the nuclear sirtuins, the mRNA expression levels of the cytosolic-nuclear sirtuin *Sirt2* were robust. High levels of *Sirt2* mRNA were detected ubiquitously throughout the different regions of the adult and developing brain examined, and in each of the peripheral tissues investigated. These results are consistent with a recent study showing *Sirt2* mRNA is expressed at high levels in the human cortex (Korner et al., 2013), and illustrate *Sirt2* mRNA is expressed at higher levels than any of the other sirtuins in the adult or developing rat brain, peripheral tissues, and also in extracts from both the neuronal and astrocyte-enriched cultures, respectively. However, there is a pronounced disconnect between the high levels of *Sirt2* mRNA expression in the embryonic and early postnatal brain, and the low to non-detectable amounts of SIRT2 protein detected by immunoblot in the same tissues. While isoforms of SIRT2 have been reported (Maxwell et al., 2011), the SIRT2 antibody we employed targets a carboxyl region of SIRT2 that is common to the different isoforms that have been characterized to date, suggesting that the differential expression we detect does not arise from the antibody we used missing an isoform-specific product lacking this epitope. This, together with the cell lineage expression results discussed below, raises the possibility that post-transcriptional mechanisms may strongly influence the expression of SIRT2 protein in the developing brain and in specific cell contexts.

There has been controversy to date over which types of neural cells express *Sirt2* in the brain. Using antisense mRNA *in situ* hybridization, as well as anti-SIRT2 immunohistochemisty, Li et al. (2007) reported strong SIRT2 expression in oligodendrocytes, but minimal to non-detectable *Sirt2* mRNA expression in neurons or astrocytes. In contrast, Maxwell et al. (2011) reported robust SIRT2 protein expression in neurons, modest expression in oligodendrocytes, and low to undetectable SIRT2 expression in astrocytes through immunohistochemical analysis. Imaoka et al. (2012) reported strong SIRT2 immunohistochemical expression in astrocytes of glioblastoma, astrocytoma, and normal human brain samples. The reasons for the discordance is not clear, but one concern with immunohistochemical studies is whether the employed antibodies label non-specific proteins in addition to their primary intended target that would cloud data interpretation. Our study assessed SIRT2 protein (as well as each of the other Sirtuin family members) by Western blotting rather than immunocytochemistry, which allowed us to focus selectively on an immunoreactive product that could be significantly and selectively suppressed by *Sirt2* targeted siRNA treatment (Supplemental Figure 1B). When combined with quantitative PCR outcomes, these immunoblot results reveal an interesting phenomenon: although *Sirt2* mRNA is clearly detected at high levels in both neuronal and astrocyte enriched cultures, SIRT2 immunoreactive protein is detected at robust levels only in the astrocyte-enriched culture extracts, with minimal or no protein detected in extracts from the neuronal cultures. While we did not examine oligodendrocytes specifically, our astrocyte-enriched cultures displayed only minor immunoreactivity for the oligodendrocyte-specific marker Ng2 relative to whole brain samples, and Ng2 was not detected in the neuronal-enriched cultures (Supplemental Figure 7). This argues against the SIRT2 protein we detect stemming from a contaminating oligodendrocytes presence in the enriched cultures. Furthermore, the developmental expression pattern for SIRT2 in cortex showed similarity to that of the GFAP (Supplemental Figure 8), indicating that the appearance of SIRT2 is largely coincident with a wave of astrogenesis in the developing postnatal cortex. A similar coincident pattern of expression was observed for the 39 kDa form of SIRT6. This coincident expression raises the possibility that the expression patterns for SIRT2 and SIRT6 in the developing brain could relate to increases in astrocyte prevalence. However, given that pronounced *Sirt2* mRNA expression was detected in both the neuronal and astrocyte enriched cultures, the relative absence of SIRT2 protein in neurons suggests that SIRT2 protein expression must be negatively regulated in neurons by post-transcriptional mechanisms that either prevent protein from being generated from expressed transcripts, or that rapidly destabilize and degrade SIRT2 protein in cultured neurons upon its synthesis.

The expression patterns of the mitochondrial sirtuins *Sirt3*, *Sirt4*, and *Sirt5* displayed a wide range of spatial and temporal divergence in the developing and adult brain, as well as considerable differences in magnitude of expression. *Sirt5* and *Sirt3* mRNA levels were found to be the second and third most abundant sirtuin transcripts in the developing and adult brain, in both the neuronal and astrocyte specific cultures, and in most of the peripheral tissues examined, respectively. The relatively higher expression levels observed for *Sirt5* and *Sirt3* mRNAs were not unexpected, as these sirtuins regulate mitochondrial homeostasis, metabolism and reactive oxygen species genesis in multiple cell types (Bell et al., 2011; reviewed in He et al., 2012; Webster et al., 2012). We found no evidence for significantly different expression levels of SIRT3, SIRT4, or SIRT5 protein in the brain tissues we assessed from rats at 24 months compared to 3 month of age, indicating an apparent lack of age-associated decrease in the brain expression of these mitochondrial sirtuins. This result contrasts with those of Zeng et al. (2014), who reported decreased SIRT3 protein levels in the aged rat auditory cortex, and Di Loreto et al. (2014) who also reported a decrease in SIRT3 protein levels in the aged mouse cortex relative to young adult under conditions of acute food and water deprivation. The reason for these differing results is unclear, but could relate to cortical subfield specific changes in SIRT3, and/or the influence of different stress conditions presented to the subjects in the studies. Because mitochondrial dysfunction is heavily implicated in aging (reviewed in Lee and Wei, 2012) and SIRT3 modulates mitochondrial metabolism and anti-oxidative defense systems (reviewed in Kincaid and Bossy-Wetzel, 2013), the preserved prevalence of SIRT3, as well as SIRT4 and SIRT5, in the aged brain confirm that drugs selectively targeting any of these sirtuins would be expected to exert effects at later stages of aging. Further, as our results indicate each of these sirtuins are expressed in both neurons and astrocytes, the same drugs would likely affect both neural cell types. Interestingly, although SIRT3 protein has been reported to be expressed two predominant forms: a 45 kDa product corresponding in mass to its precursor form, and a 28 kDa product corresponding to its form following import into mitochondria after processing by mitochondrial processing peptidase (Onyango et al., 2002), we could only verify the 28 kDa product recognized with the antibody we employed as originating from Sirt3 (Supplemental Figure 1C). It is not clear whether a 45 kDa SIRT3 product is also present in brain that is masked in the SIRT3-null mouse brain by a cross-reactive co-migrating non-specific protein. As such, we only report the protein expression profile for the processed form of SIRT3, which could conceivably differ from the 45 kDa form that has been detected in the nucleus (Iwahara et al., 2012). Additional studies will be required to resolve this possibility. Finally, in contrast to the relatively high expression levels observed for *Sirt3* and *Sirt5*, *Sirt4* was generally the least expressed member of the sirtuin family in all of the adult tissues and cell types examined. The notably higher level of *Sirt4* mRNA expression in late embryonic stage cortex and hippocampus compared to perinatal or adult brain regions suggest *Sirt4* may be more prominently expressed during embryogenesis, and that a closer examination of *Sirt4* during embryonic brain development may be warranted.

In sum, this study illustrates sirtuin family members display differential expression patterns within the developing and adult brain at both the mRNA and protein levels, with specific family members displaying preferential expression in either astrocytes or neurons. Our results indicate that mRNA for each of the seven sirtuins is expressed in the brain throughout development. In all brain regions examined during development, *Sirt2* was the most prevalently expressed sirtuin, and *Sirt4* was the least expressed member of the family. No significant decreases in the expression of any sirtuin member were observed in any brain region between the 24 month old and 3 month old rats. However, the mRNA expression pattern for specific sirtuins did not always parallel its corresponding protein expression pattern. This was most evident for SIRT2, where protein expression was largely absent at early developmental stages despite the presence of pronounced mRNA expression. This differential expression was also noted in cell type specificity, where *Sirt2* mRNA was detected in both astrocytes and neurons whereas SIRT2 protein was selectively observed in astrocytes. Sirt5 also displayed similar differential cell type expression, as SIRT5 protein was preferentially observed in neurons despite its mRNA being present in both astrocytes and neurons. Collectively, these results add to our knowledge of the regulation of sirtuin expression within the brain at different developmental stages, and provide information on which specific sirtuins could potentially be targeted pharmacologically in the brain at times ranging from perinatal development to 24 months of age. These data also provide evidence that specific sirtuins could potentially be targeted to achieve cell-type preferential effects within the brain.

### Conflict of interest statement

The authors declare that the research was conducted in the absence of any commercial or financial relationships that could be construed as a potential conflict of interest.
