# Supplementary material for: Differential expression of sirtuin family members in the developing, adult, and aged rat brain
Source: Front Aging Neurosci. 2014 Dec 18;6:333. doi: 10.3389/fnagi.2014.00333 (PMC4270178; doi:10.3389/fnagi.2014.00333)
Supplement: Supplementary file 1 [file Presentation1.PDF]

Supplemental Figure 1

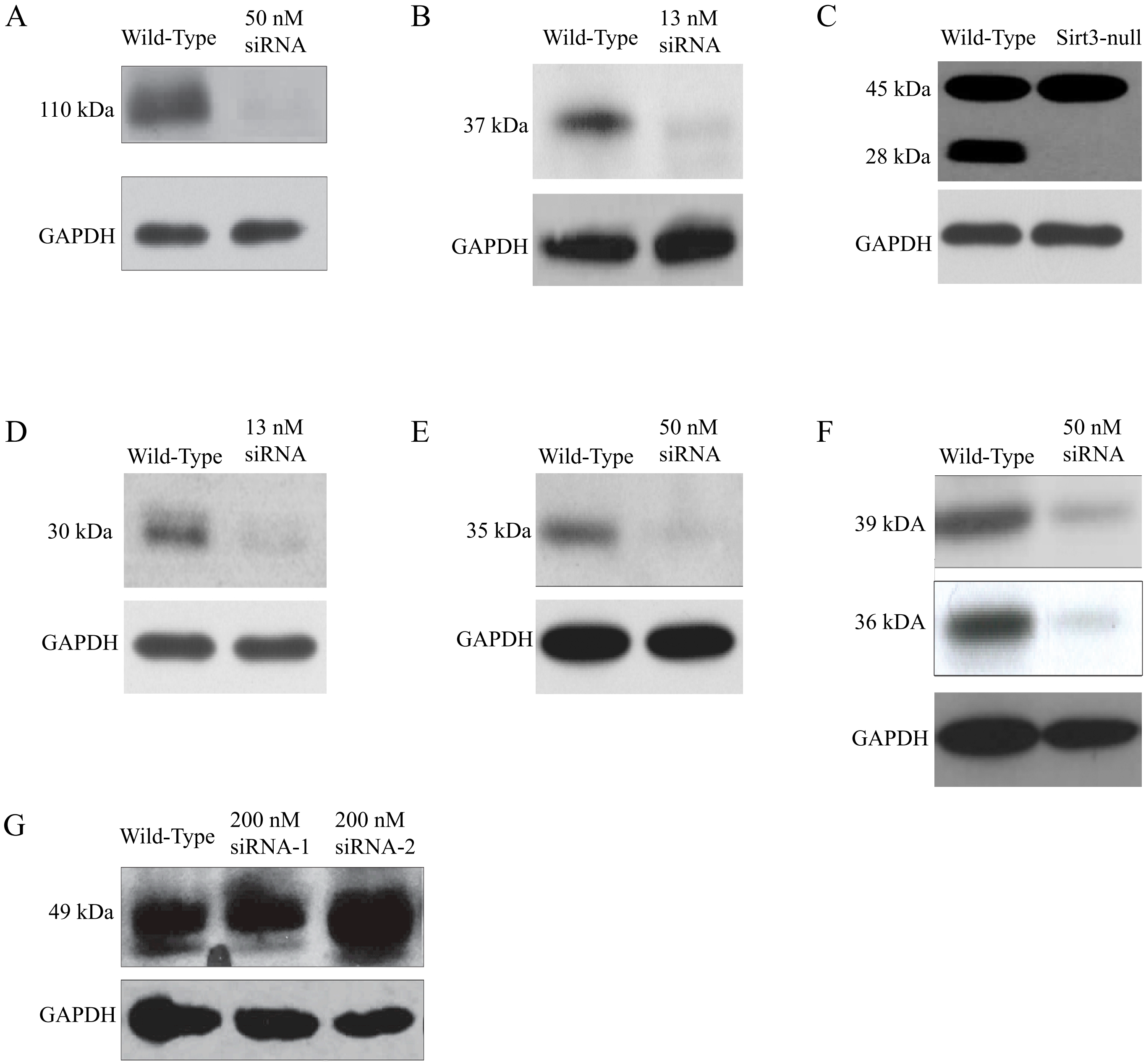

Figure S1. Validation of Sirtuin Antibodies Specificity Using siRNA. Representative Western blots showing the specificity of the immunoreactive products in rat whole cell PC12 lysates by using sirtuin-specific siRNA in **(A)** SIRT1 **(B)** SIRT2 (**D)** SIRT4 (**E)** SIRT5 (**F)** SIRT6 **(G)** SIRT7 and **(C)** in SIRT3-KO mice lacking SIRT3 expression. GAPDH immunoreactivity is shown as a protein loading control.

Supplemental Figure 2

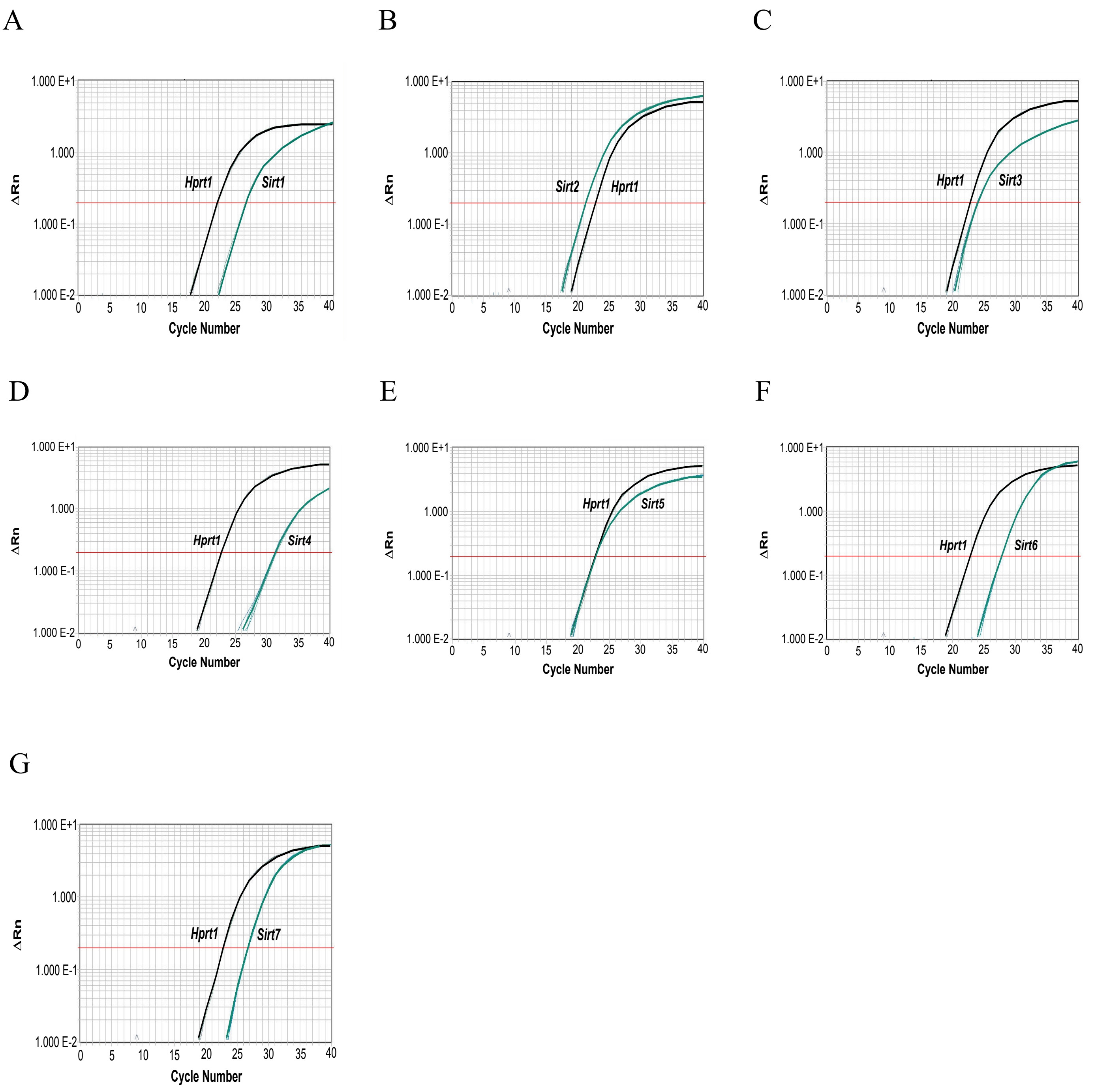

Figure S2. **Representative Amplification Traces of Sirtuin Gene Expression Levels Relative to *Hprt1*.** Representative Quantitative Real-Time PCR plots are shown for the mRNA expression levels of **(A)** *Sirt1* **(B)** *Sirt2* **(C)** *Sirt3* **(D)** *Sirt4* **(E)** *Sirt5* **(F)** *Sirt6* **(G)** *Sirt7* in the rat whole brain homogenates, the amplification profile of the reference transcript *Hprt1* is also indicated.

Supplemental Figure 3

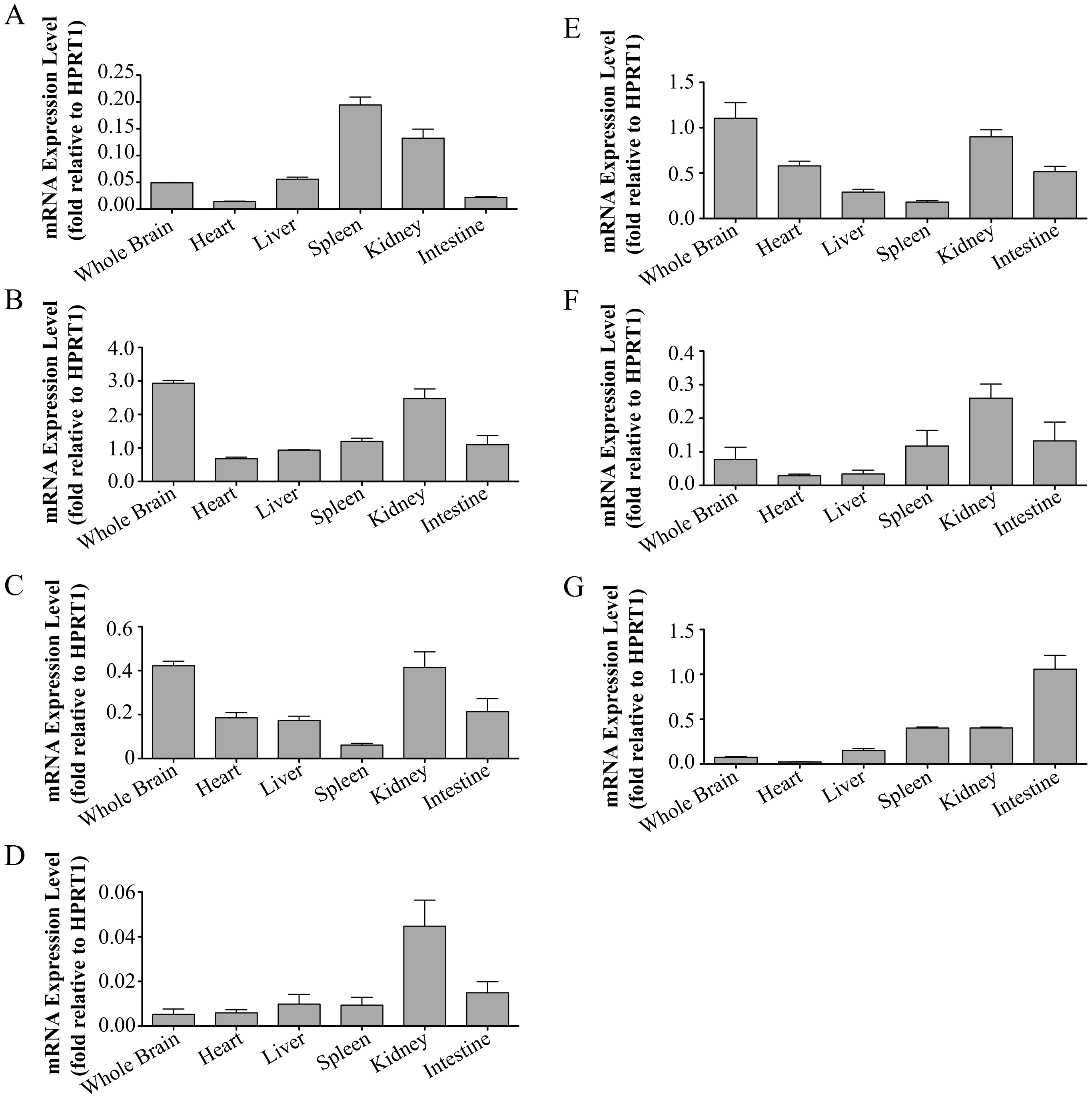

Figure S3. Sirtuins Display Differential Gene Expression Patterns In Adult Peripheral Tissues. Histograms showing the mean and SEM of the mRNA expression levels for each individual sirtuin in the indicated peripheral tissues and whole brain as determined by qRT-PCR. The y-axis of each histogram shows the relative mRNA expression levels of **(A)** *Sirt1* **(B)** *Sirt2* **(C)** *Sirt3* **(D)** *Sirt4* **(E)** *Sirt5* **(F)** *Sirt6* **(G)** *Sirt7* normalized to the *Hprt1* reference gene. Data shown are based on the linear conversion of delta CT values for each sample (n=3 independent subjects done in quadruplicate). Statistical comparisons between the mRNA expression levels for each sirtuin in these tissues is presented in Supplemental Table 4.

Supplemental Figure 4

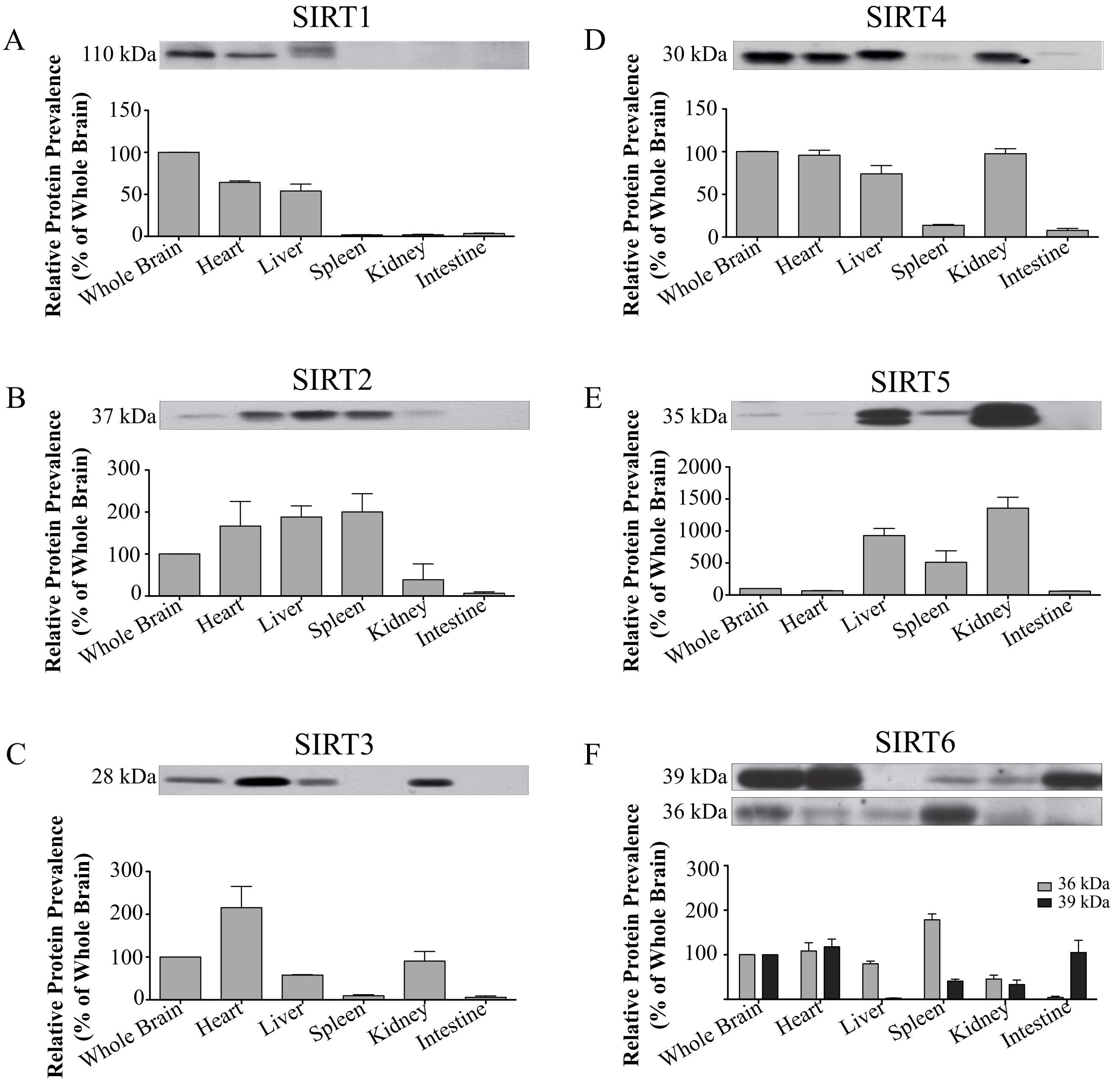

Figure S4. Sirtuin Protein Expression Patterns in Adult Rat Peripheral Tissues Relative To Brain. Representative Western blots showing the immunoreactive prevalence of **(A)** SIRT1 **(B)** SIRT2 (**C)** SIRT3 (**D)** SIRT4 (**E)** SIRT5 (**F)** SIRT6 in the indicated peripheral tissues of adult rats. The histogram in each panel shows the densitometric mean and SEM for the indicated sirtuin. For these calculations, Coomassie blue staining was used as a protein loading control due to variability in GAPDH expression within the peripheral tissues (n=3 independent subjects). The expression levels for each sirtuin in these peripheral tissues are presented relative to their expression level in brain. Statistical comparisons between sirtuin protein expression levels in these adult tissues are presented in Supplemental Table 5.

Supplemental Figure 5

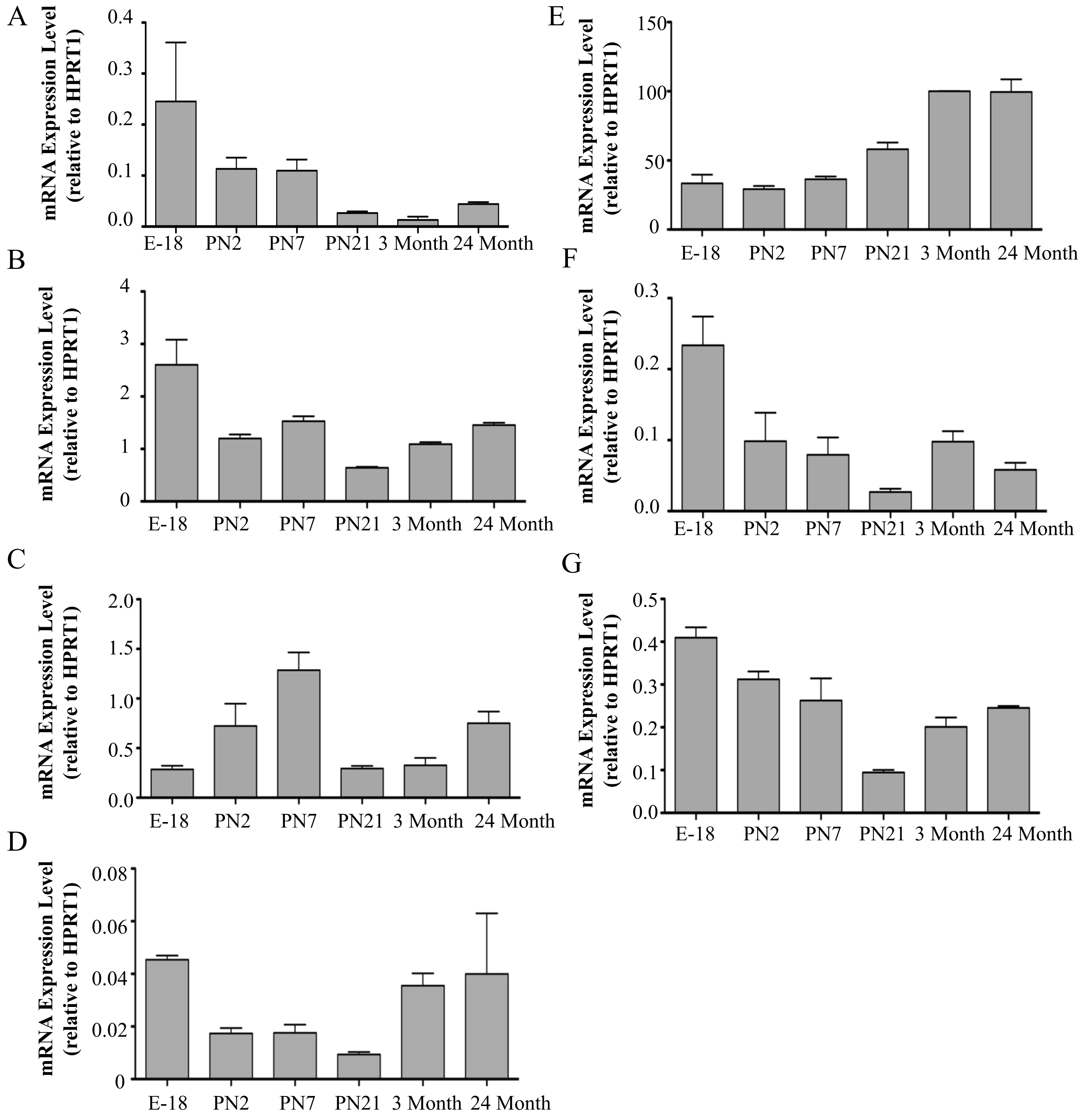

Figure S5. Sirtuins Display Differential Gene Expression Patterns During Liver Development in Rat. Histograms showing the mean and SEM of the mRNA expression levels for each individual sirtuin as determined by qRT-PCR at the indicated stages of liver development (E18 - 3 months) and in rats at 24 months of age. The y-axis of each histogram shows the relative expression levels of **(A)** *Sirt1* **(B)** *Sirt2* (**C**) *Sirt3* (**D**) *Sirt4* (**E**) *Sirt5* (**F**) *Sirt6* (**G**) *Sirt7* relative to the *Hprt1* reference gene. Data shown are based on the linear conversion of delta CT values for each sample (n=3 independent subjects done in quadruplicate). Statistical comparisons for the expression levels of each sirtuin for the developmental times shown are presented in Supplemental Table 12.

Supplemental Figure 6

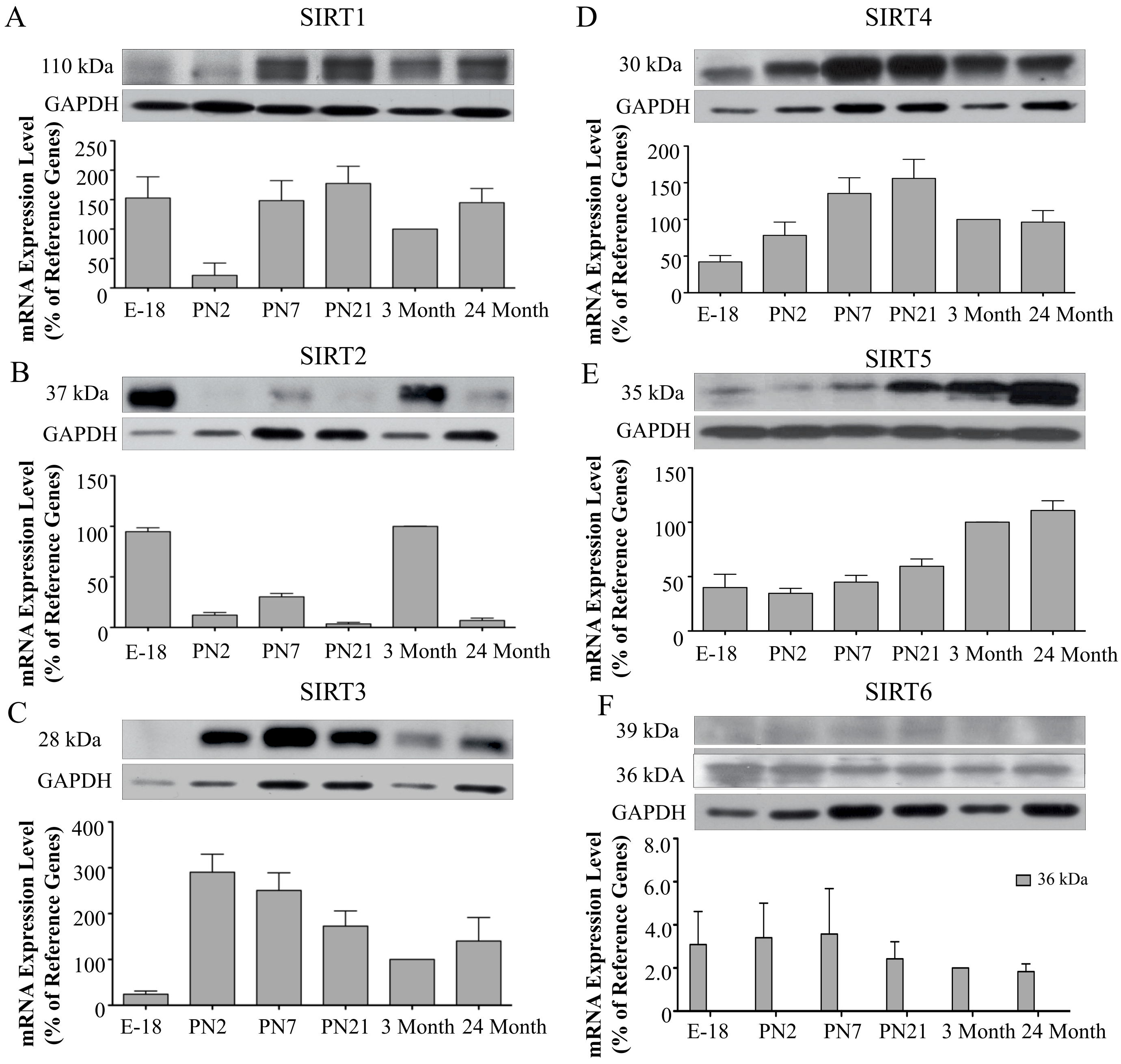

Figure S6. Sirtuins Display Differential Protein Expression Patterns During Liver Development in Rat. Representative Western blots showing the immunoreactive prevalence of sirtuins at the indicated stages of liver development (E18 - 3 months) and in rats at 24 months of age. The y-axis of each histogram shows the relative expression levels of **(A)** *Sirt1* **(B)** *Sirt2* **(C)** *Sirt3* **(D)** *Sirt4* **(E)** *Sirt5* **(F)** *Sirt6* **(G)** Sirt7 for the indicated stages of development. The histograms in each panel show the densitometric mean and SEM for each specific sirtuin normalized to its corresponding GAPDH loading control. (n=3 independent subjects). Statistical comparisons for the expression levels of each sirtuin for the developmental times shown are presented in Supplemental Table 13.

Supplemental Figure 7

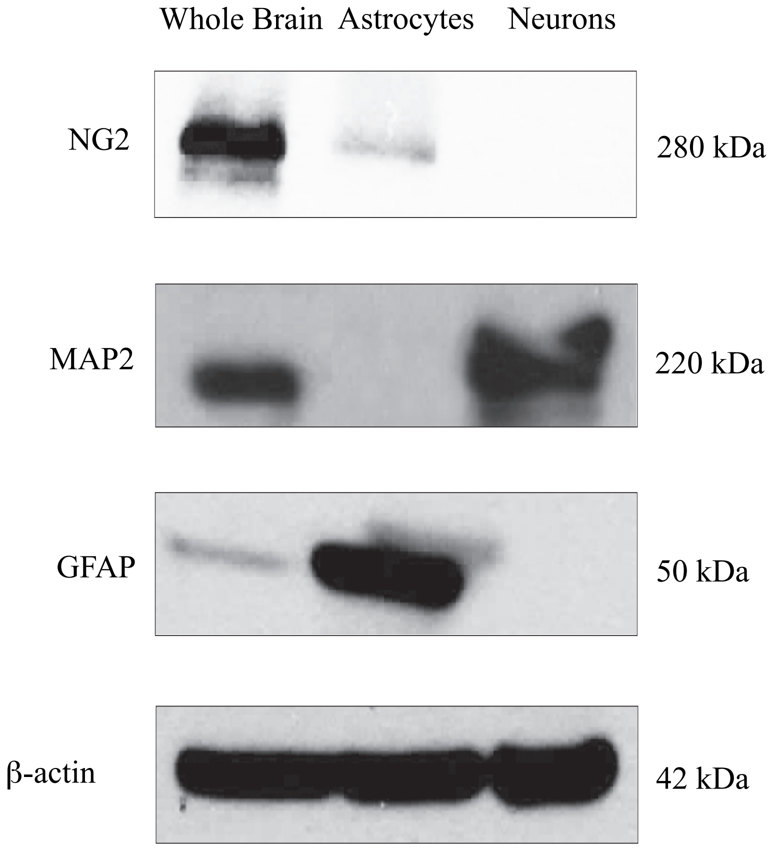

Figure S7. Assessment of Purity of Neuronal and Astrocyte Cultures. Representative Western blot showing the immunoreactivity of astrocyte-specific (GFAP), neuron-specific (MAP2) and oligodendocyte-specific (NG2) markers in adult rat brain homogenate and in cultured rat neuronal and astrocytic whole cell lysates. Beta-actin immunoreactivity is shown as a protein loading control. No astrocyte or neuronal contamination is visible in either of the cell lysates. No oligodendrocyte contamination is visible in neuronal cell lysates and minimal is observed in astrocyte lysate.

Supplemental Figure 8

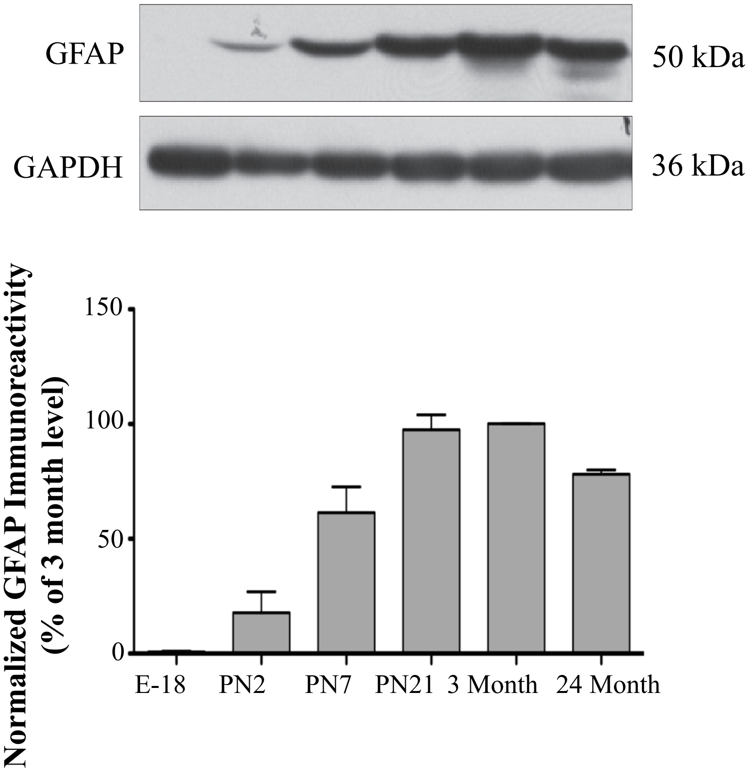

Figure S8. GFAP Protein Expression During Cortical Development in Rat. Representative Western blot showing the immunoreactive prevalence of GFAP at the indicated stages of cortex development (E18 - 3 months) and in rats at 24 months of age. The histogram shows the densitometric mean and SEM for GFAP expression is normalized to its corresponding GAPDH loading control. (n=3 independent subjects).

Table 1: Comparison of sirtuin mRNA expression levels in the whole adult rat brain

Gene	Summary
Sirt1 vs. *Sirt2*	***
Sirt1 vs. *Sirt3*	*
Sirt1 vs. *Sirt4*	ns
Sirt1 vs. *Sirt5*	***
Sirt1 vs. *Sirt6*	ns
Sirt1 vs. *Sirt7*	ns
Sirt2 vs. *Sirt3*	***
Sirt2 vs. *Sirt4*	***
Sirt2 vs. *Sirt5*	***
Sirt2 vs. *Sirt6*	***
Sirt2 vs. *Sirt7*	***
Sirt3 vs. *Sirt4*	*
Sirt3 vs. Sirt5	***
Sirt3 vs. *Sirt6*	ns
Sirt3 vs. *Sirt7*	ns
Sirt4 vs. *Sirt5*	***
Sirt4 vs. *Sirt6*	ns
Sirt4 vs. *Sirt7*	ns
Sirt5 vs. *Sirt6*	***
Sirt5 vs. *Sirt7*	***
Sirt6 vs. *Sirt7*	ns

Asterisks denote significance at *p < 0.05, **p <0.01 and ***p < 0.001, One-way ANOVA, with Tukey post-hoc test (n=3). ns designates no statistical difference in expression

Table 2: Comparison of sirtuin mRNA expression in 3 month-old rat brain regions

	Sirt1	*Sirt2*	*Sirt3*	*Sirt4*	*Sirt5*	*Sirt6*	*Sirt7*
Cortex vs. Hippocampus	ns	ns	ns	ns	ns	ns	ns
Cortex vs. Cerebellum	ns	ns	ns	ns	ns	ns	ns
Cortex vs. Brain Stem	ns	**	**	ns	ns	ns	ns
Cortex vs. Spinal Cord	ns	***	*	***	**	ns	ns
Cortex vs. Striatum	ns	ns	*	ns	*	ns	ns
Cortex vs. Olfactory Bulb	ns	ns	ns	ns	**	ns	ns
Hippocampus vs. Cerebellum	*	ns	*	ns	ns	ns	ns
Hippocampus vs. Brain Stem	ns	*	ns	ns	ns	ns	ns
Hippocampus vs. Spinal Cord	ns	***	ns	***	ns	ns	ns
Hippocampus vs. Striatum	ns	ns	ns	*	ns	ns	ns
Hippocampus vs. Olfactory Bulb	ns	ns	ns	ns	*	**	ns
Cerebellum vs. Brain Stem	ns	**	***	ns	ns	ns	ns
Cerebellum vs. Spinal Cord	**	***	***	***	**	ns	ns
Cerebellum vs. Striatum	*	ns	**	ns	*	ns	ns
Cerebellum vs. Olfactory Bulb	ns	ns	ns	ns	**	*	ns
Brain Stem vs. Spinal Cord	*	**	ns	***	ns	ns	ns
Brain Stem vs. Striatum	ns	ns	ns	*	ns	ns	ns
Brain Stem vs. Olfactory bulb	ns	*	*	ns	*	**	ns
Spinal Cord vs. Striatum	ns	***	ns	***	ns	ns	ns
Spinal Cord vs. Olfactory Bulb	ns	***	ns	***	ns	ns	ns
Striatum vs. Olfactory Bulb	ns	ns	ns	ns	ns	ns	ns

Asterisks denote significance at *p < 0.05, **p <0.01 and ***p < 0.001, One-way ANOVA, with Tukey post-hoc test (n=3). ns designates no statistical difference in expression

Table 3: Comparison of sirtuin protein expression in 3 month-old rat brain regions

	SIRT1	SIRT2	SIRT3	SIRT4	SIRT5	SIRT6 36kDa	SIRT6 39kDa	SIRT7
Cortex vs. Hippocampus	ns	ns	ns	ns	ns	ns	ns	N/A
Cortex vs. Cerebellum	*	ns	ns	ns	*	ns	ns	N/A
Cortex vs. Brain Stem	ns	ns	ns	ns	ns	ns	ns	N/A
Cortex vs. Spinal Cord	ns	*	ns	ns	ns	ns	ns	N/A
Cortex vs. Striatum	ns	ns	ns	ns	ns	ns	ns	N/A
Cortex vs. Olfactory Bulb	ns	ns	ns	ns	ns	ns	ns	N/A
Hippocampus vs. Cerebellum	ns	ns	*	ns	*	ns	ns	N/A
Hippocampus vs. Brain Stem	ns	ns	ns	ns	ns	ns	ns	N/A
Hippocampus vs. Spinal Cord	ns	ns	ns	ns	ns	ns	ns	N/A
Hippocampus vs. Striatum	ns	ns	ns	ns	ns	ns	ns	N/A
Hippocampus vs. Olfactory Bulb	ns	ns	ns	ns	ns	ns	ns	N/A
Cerebellum vs. Brain Stem	ns	ns	ns	ns	ns	ns	ns	N/A
Cerebellum vs. Spinal Cord	**	*	ns	ns	ns	ns	ns	N/A
Cerebellum vs. Striatum	*	ns	ns	*	ns	ns	ns	N/A
Cerebellum vs. Olfactory Bulb	ns	ns	ns	ns	*	ns	ns	N/A
Brain Stem vs. Spinal Cord	ns	ns	ns	ns	ns	ns	ns	N/A
Brain Stem vs. Striatum	ns	ns	ns	ns	ns	ns	ns	N/A
Brain Stem vs. Olfactory Bulb	ns	ns	ns	ns	ns	ns	ns	N/A
Spinal Cord vs. Striatum	ns	ns	ns	ns	ns	ns	ns	N/A
Spinal Cord vs. Olfactory Bulb	ns	*	ns	ns	ns	ns	ns	N/A
Striatum vs. Olfactory Bulb	ns	ns	ns	ns	ns	ns	ns	N/A

Asterisks denote significance at *p < 0.05, **p <0.01 and ***p < 0.001, One-way ANOVA, with Tukey post-hoc test (n=3). ns designates no statistical difference in expression

Table 4: Comparison of sirtuin mRNA expression in 3 month-old rat peripheral regions

	Sirt1	*Sirt2*	*Sirt3*	*Sirt4*	*Sirt5*	*Sirt6*	*Sirt7*
Whole Brain vs. Heart	ns	***	*	ns	**	ns	ns
Whole Brain vs. Liver	ns	***	*	ns	***	ns	ns
Whole Brain vs. Spleen	***	***	***	ns	***	ns	*
Whole Brain vs. Kidney	***	ns	ns	**	ns	*	*
Whole Brain vs. Intestine	ns	***	*	ns	**	ns	***
Heart vs. Liver	ns	ns	ns	ns	ns	ns	ns
Heart vs. Spleen	***	ns	ns	ns	ns	ns	*
Heart vs. Kidney	***	***	*	**	ns	*	*
Heart vs. Intestine	ns	ns	ns	ns	ns	ns	***
Liver vs. Spleen	***	ns	ns	ns	ns	ns	ns
Liver vs. Kidney	***	***	*	*	**	*	ns
Liver vs. Intestine	ns	ns	ns	ns	ns	ns	***
Spleen vs. Kidney	**	**	***	**	***	ns	ns
Spleen vs. Intestine	***	ns	ns	ns	ns	ns	***
Kidney vs. Intestine	***	***	*	*	ns	ns	***

Asterisks denote significance at *p < 0.05, **p <0.01 and ***p < 0.001, One-way ANOVA, with Tukey post-hoc test (n=3). ns designates no statistical difference in expression

Table 5: Comparison of sirtuin protein expression in 3 month-old rat peripheral regions

	SIRT1	SIRT2	SIRT3	SIRT4	SIRT5	SIRT6 36kDa	SIRT6 39kDa	SIRT7
Whole Brain vs. Heart	***	ns	*	ns	ns	ns	ns	N/A
Whole Brain vs. Liver	***	ns	ns	*	**	ns	**	N/A
Whole Brain vs. Spleen	***	ns	ns	***	ns	**	ns	N/A
Whole Brain vs. Kidney	***	ns	ns	ns	***	*	*	N/A
Whole Brain vs. Intestine	***	ns	ns	***	ns	***	ns	N/A
Heart vs. Liver	ns	ns	**	ns	**	ns	***	N/A
Heart vs. Spleen	***	ns	***	***	ns	**	*	N/A
Heart vs. Kidney	***	ns	*	ns	***	*	*	N/A
Heart vs. Intestine	***	ns	***	***	ns	***	ns	N/A
Liver vs. Spleen	***	ns	ns	***	ns	***	ns	N/A
Liver vs. Kidney	***	ns	ns	ns	ns	ns	ns	N/A
Liver vs. Intestine	***	*	ns	***	**	**	**	N/A
Spleen vs. Kidney	ns	ns	ns	***	**	***	ns	N/A
Spleen vs. Intestine	ns	*	ns	ns	ns	***	ns	N/A
Kidney vs. Intestine	ns	ns	ns	***	***	ns	*	N/A

Asterisks denote significance at *$p < 0.05$, **$p < 0.01$ and ***$p < 0.001$, One-way ANOVA, with Tukey post-hoc test (n=3). ns designates no statistical difference in expression

Table 6: Comparison of sirtuin mRNA expression during cortical development

	Sirt1	*Sirt2*	*Sirt3*	*Sirt4*	*Sirt5*	*Sirt6*	*Sirt7*
E-18 vs. PN2	***	**	ns	**	**	ns	***
E-18 vs. PN7	***	***	ns	***	***	**	***
E-18 vs. PN21	***	ns	ns	***	***	**	***
E-18 vs. 3 months	***	***	ns	***	***	**	***
E-18 vs. 24 months	***	***	ns	***	***	**	***
PN2 vs. PN7	ns	ns	ns	ns	*	ns	*
PN2 vs. PN21	ns	*	ns	ns	***	ns	**
PN2 vs. 3 months	*	ns	ns	ns	**	ns	**
PN2 vs. 24 months	**	ns	ns	ns	**	ns	**
PN7 vs. PN21	ns	*	ns	ns	ns	ns	ns
PN7 vs. 3 months	ns	ns	ns	ns	ns	ns	ns
PN7 vs. 24 months	ns	ns	ns	ns	ns	ns	ns
PN21 vs. 3 months	ns	***	ns	ns	ns	ns	ns
PN21 vs. 24 months	ns	**	ns	ns	ns	ns	ns
3 months vs. 24 months	ns	ns	ns	ns	ns	ns	ns

Asterisks denote significance at *$p < 0.05$, **$p < 0.01$ and ***$p < 0.001$, One-way ANOVA, with Tukey post-hoc test (n=3). ns designates no statistical difference in expression

Table 7: Comparison of sirtuin protein expression during cortical development

	SIRT1	SIRT2	SIRT3	SIRT4	SIRT5	SIRT6 36kDa	SIRT6 39 kDa	SIRT7
E-18 vs. PN2	**	ns	ns	ns	ns	**	ns	N/A
E-18 vs. PN7	***	ns	ns	ns	ns	***	ns	N/A
E-18 vs. PN21	***	***	**	ns	ns	***	ns	N/A
E-18 vs. 3 months	***	***	***	ns	ns	***	ns	N/A
E-18 vs. 24 months	***	***	**	ns	ns	***	ns	N/A
PN2 vs. PN7	ns	ns	ns	ns	ns	ns	ns	N/A
PN2 vs. PN21	ns	***	**	ns	ns	*	ns	N/A
PN2 vs. 3 months	ns	***	***	ns	ns	*	ns	N/A
PN2 vs. 24 months	ns	***	**	ns	ns	*	ns	N/A
PN7 vs. PN21	ns	***	*	ns	ns	ns	ns	N/A
PN7 vs. 3 months	ns	***	**	ns	ns	ns	ns	N/A
PN7 vs. 24 months	ns	***	*	ns	ns	ns	ns	N/A
PN21 vs. 3 months	ns	ns	ns	ns	ns	ns	ns	N/A
PN21 vs. 24 months	ns	ns	ns	ns	ns	ns	ns	N/A
3 months vs. 24 months	ns	ns	ns	ns	ns	ns	ns	N/A

Asterisks denote significance at *$p < 0.05$, **$p < 0.01$ and ***$p < 0.001$, One-way ANOVA, with Tukey post-hoc test (n=3). ns designates no statistical difference in expression

Table 8: Comparison of sirtuin mRNA expression during hippocampal development

	Sirt1	*Sirt2*	*Sirt3*	*Sirt4*	*Sirt5*	*Sirt6*	*Sirt7*
E-18 vs. PN2	ns	***	ns	***	***	***	***
E-18 vs. PN7	ns	***	*	***	***	***	***
E-18 vs. PN21	ns	***	ns	***	***	***	***
E-18 vs. 3 months	ns	***	ns	***	***	***	***
E-18 vs. 24 months	ns	***	ns	***	***	***	***
PN2 vs. PN7	ns	ns	ns	ns	*	ns	***
PN2 vs. PN21	ns	***	ns	ns	**	ns	***
PN2 vs. 3 months	ns	ns	ns	ns	***	ns	***
PN2 vs. 24 months	ns	ns	ns	ns	*	*	***
PN7 vs. PN21	ns	***	*	ns	ns	ns	ns
PN7 vs. 3 months	ns	ns	ns	ns	ns	ns	ns
PN7 vs. 24 months	ns	ns	*	ns	ns	ns	ns
PN21 vs. 3 months	ns	***	ns	ns	ns	ns	ns
PN21 vs. 24 months	ns	***	ns	ns	ns	ns	ns
3 months vs. 24 months	ns	ns	ns	ns	ns	ns	ns

Asterisks denote significance at *$p < 0.05$, **$p < 0.01$ and ***$p < 0.001$, One-way ANOVA, with Tukey post-hoc test (n=3). ns designates no statistical difference in expression

Table 9: Comparison of sirtuin protein expression during hippocampal development

	SIRT1	SIRT2	SIRT3	SIRT4	SIRT5	SIRT6 36kDa	SIRT6 39 kDa	SIRT7
E-18 vs. PN2	ns	ns	ns	ns	ns	ns	ns	N/A
E-18 vs. PN7	*	ns	ns	ns	ns	ns	ns	N/A
E-18 vs. PN21	**	***	**	ns	ns	*	ns	N/A
E-18 vs. 3 months	***	***	***	ns	ns	*	ns	N/A
E-18 vs. 24 months	***	***	***	ns	ns	*	ns	N/A
PN2 vs. PN7	ns	ns	ns	ns	ns	ns	ns	N/A
PN2 vs. PN21	*	***	*	ns	ns	ns	ns	N/A
PN2 vs. 3 months	*	***	***	ns	ns	ns	ns	N/A
PN2 vs. 24 months	**	***	***	ns	ns	ns	ns	N/A
PN7 vs. PN21	ns	***	ns	ns	ns	ns	ns	N/A
PN7 vs. 3 months	ns	***	***	ns	ns	ns	ns	N/A
PN7 vs. 24 months	ns	***	**	ns	ns	ns	ns	N/A
PN21 vs. 3 months	ns	ns	ns	ns	ns	ns	ns	N/A
PN21 vs. 24 months	ns	ns	ns	ns	ns	ns	ns	N/A
3 months vs. 24 months	ns	ns	ns	ns	ns	ns	ns	N/A

Asterisks denote significance at *$p < 0.05$, **$p < 0.01$ and ***$p < 0.001$, One-way ANOVA, with Tukey post-hoc test (n=3). ns designates no statistical difference in expression

Table 10: Comparison of sirtuin mRNA expression during cerebellar development

	Sirt1	*Sirt2*	*Sirt3*	*Sirt4*	*Sirt5*	*Sirt6*	*Sirt7*
E-18 vs. PN2	ns	ns	ns	***	ns	***	ns
E-18 vs. PN7	***	***	ns	***	ns	***	ns
E-18 vs. PN21	ns	*	ns	***	**	***	*
E-18 vs. 3 months	**	*	ns	***	**	***	**
E-18 vs. 24 months	*	ns	ns	***	*	***	**
PN2 vs. PN7	***	***	ns	ns	ns	ns	*
PN2 vs. PN21	*	ns	ns	ns	ns	ns	ns
PN2 vs. 3 months	ns	**	ns	ns	*	ns	ns
PN2 vs. 24 months	ns	*	ns	ns	ns	ns	ns
PN7 vs. PN21	***	ns	ns	ns	ns	ns	**
PN7 vs. 3 months	***	***	ns	ns	*	ns	**
PN7 vs. 24 months	***	***	ns	ns	ns	ns	**
PN21 vs. 3 months	***	***	ns	ns	ns	ns	ns
PN21 vs. 24 months	**	***	ns	ns	ns	ns	ns
3 months vs. 24 months	ns	ns	ns	ns	ns	ns	ns

Asterisks denote significance at *p < 0.05, **p <0.01 and ***p < 0.001, One-way ANOVA, with Tukey post-hoc test (n=3). ns designates no statistical difference in expression

Table 11: Comparison of sirtuin protein expression during cerebellar development

	**SIRT1**	**SIRT2**	**SIRT3**	**SIRT4**	**SIRT5**	**SIRT6 36kDa**	**SIRT6 39 kDa**	**SIRT7**
E-18 vs. PN2	ns	ns	ns	ns	ns	ns	ns	N/A
E-18 vs. PN7	ns	ns	ns	ns	ns	ns	ns	N/A
E-18 vs. PN21	ns	***	ns	ns	ns	ns	**	N/A
E-18 vs. 3 months	ns	***	ns	ns	*	ns	**	N/A
E-18 vs. 24 months	ns	***	*	ns	**	ns	**	N/A
PN2 vs. PN7	ns	ns	ns	ns	ns	ns	ns	N/A
PN2 vs. PN21	ns	***	ns	ns	ns	ns	**	N/A
PN2 vs. 3 months	*	***	ns	ns	ns	ns	**	N/A
PN2 vs. 24 months	*	***	ns	ns	ns	ns	**	N/A
PN7 vs. PN21	ns	***	ns	ns	ns	ns	*	N/A
PN7 vs. 3 months	*	***	ns	ns	ns	ns	**	N/A
PN7 vs. 24 months	*	***	**	ns	*	ns	*	N/A
PN21 vs. 3 months	ns	ns	ns	ns	*	ns	ns	N/A
PN21 vs. 24 months	ns	ns	ns	ns	**	ns	ns	N/A
3 months vs. 24 months	ns	ns	ns	ns	ns	ns	ns	N/A

Asterisks denote significance at $*p < 0.05$, $**p < 0.01$ and $***p < 0.001$, One-way ANOVA, with Tukey post-hoc test (n=3). ns designates no statistical difference in expression

Table 12: Comparison of sirtuin mRNA expression during liver development

	Sirt1	*Sirt2*	*Sirt3*	*Sirt4*	*Sirt5*	*Sirt6*	*Sirt7*
E-18 vs. PN2	ns	**	ns	ns	ns	*	ns
E-18 vs. PN7	ns	*	**	ns	ns	*	*
E-18 vs. PN21	ns	***	ns	*	*	**	***
E-18 vs. 3 months	ns	**	ns	ns	***	*	**
E-18 vs. 24 months	ns	*	ns	ns	***	*	**
PN2 vs. PN7	ns	ns	ns	ns	ns	ns	ns
PN2 vs. PN21	ns	ns	ns	ns	*	ns	***
PN2 vs. 3 months	ns	ns	ns	ns	***	ns	ns
PN2 vs. 24 months	ns	ns	ns	ns	***	ns	ns
PN7 vs. PN21	ns	ns	**	ns	ns	ns	**
PN7 vs. 3 months	ns	ns	**	ns	***	ns	ns
PN7 vs. 24 months	ns	ns	ns	ns	***	ns	ns
PN21 vs. 3 months	ns	ns	ns	ns	***	ns	ns
PN21 vs. 24 months	ns	ns	ns	ns	***	ns	*
3 months vs. 24 months	ns	ns	ns	ns	ns	ns	ns

Asterisks denote significance at *$p < 0.05$, **$p < 0.01$ and ***$p < 0.001$, One-way ANOVA, with Tukey post-hoc test (n=3). ns designates no statistical difference in expression

Table 13: Comparison of sirtuin protein expression during liver development

	SIRT1	SIRT2	SIRT3	SIRT4	SIRT5	SIRT6 36kDa	SIRT6 39 kDa	SIRT7
E-18 vs. PN2	*	***	**	ns	ns	ns	N/A	N/A
E-18 vs. PN7	ns	***	**	ns	ns	ns	N/A	N/A
E-18 vs. PN21	ns	***	ns	ns	ns	ns	N/A	N/A
E-18 vs. 3 months	ns	ns	ns	**	**	ns	N/A	N/A
E-18 vs. 24 months	ns	***	ns	***	***	ns	N/A	N/A
PN2 vs. PN7	ns	**	ns	ns	ns	ns	N/A	N/A
PN2 vs. PN21	*	ns	ns	ns	ns	ns	N/A	N/A
PN2 vs. 3 months	ns	***	*	***	***	ns	N/A	N/A
PN2 vs. 24 months	ns	ns	ns	***	***	ns	N/A	N/A
PN7 vs. PN21	ns	***	ns	ns	ns	ns	N/A	N/A
PN7 vs. 3 months	ns	***	ns	**	**	ns	N/A	N/A
PN7 vs. 24 months	ns	***	ns	***	***	ns	N/A	N/A
PN21 vs. 3 months	ns	***	ns	*	*	ns	N/A	N/A
PN21 vs. 24 months	ns	ns	ns	**	**	ns	N/A	N/A
3 months vs. 24 months	ns	***	ns	ns	ns	ns	N/A	N/A

Asterisks denote significance at *$p < 0.05$, **$p < 0.01$ and ***$p < 0.001$, One-way ANOVA, with Tukey post-hoc test (n=3). ns designates no statistical difference in expression
